# Routine Multiplex Mutational Profiling of Melanomas Enables Enrollment in Genotype-Driven Therapeutic Trials

**DOI:** 10.1371/journal.pone.0035309

**Published:** 2012-04-20

**Authors:** Christine M. Lovly, Kimberly Brown Dahlman, Laurel E. Fohn, Zengliu Su, Dora Dias-Santagata, Donna J. Hicks, Donald Hucks, Elizabeth Berry, Charles Terry, MarKeesa Duke, Yingjun Su, Tammy Sobolik-Delmaire, Ann Richmond, Mark C. Kelley, Cindy L. Vnencak-Jones, A. John Iafrate, Jeffrey Sosman, William Pao

**Affiliations:** 1 Division of Hematology-Oncology, Department of Medicine, Vanderbilt-Ingram Cancer Center, Vanderbilt University School of Medicine, Nashville, Tennessee, United States of America; 2 Department of Cancer Biology, Vanderbilt-Ingram Cancer Center, Vanderbilt University School of Medicine, Nashville, Tennessee, United States of America; 3 Translational Research Laboratory, Department of Pathology, Massachusetts General Hospital and Harvard Medical School, Boston, Massachusetts, United States of America; 4 Vanderbilt-Ingram Cancer Center, Vanderbilt University Medical Center, Nashville, Tennessee, United States of America; 5 Department of Cancer Biology, Department of Veterans Affairs, Vanderbilt-Ingram Cancer Center, Vanderbilt University School of Medicine, Nashville, Tennessee, United States of America; 6 Division of Surgical Oncology, Vanderbilt-Ingram Cancer Center, Vanderbilt University School of Medicine, Nashville, Tennessee, United States of America; 7 Departments of Pathology, Microbiology, Immunology and Pediatrics, Vanderbilt University Medical Center, Nashville, Tennessee, United States of America; The Moffitt Cancer Center & Research Institute, United States of America

## Abstract

**Purpose:**

Knowledge of tumor mutation status is becoming increasingly important for the treatment of cancer, as mutation-specific inhibitors are being developed for clinical use that target only sub-populations of patients with particular tumor genotypes. Melanoma provides a recent example of this paradigm. We report here development, validation, and implementation of an assay designed to simultaneously detect 43 common somatic point mutations in 6 genes (*BRAF, NRAS, KIT, GNAQ, GNA11*, and *CTNNB1*) potentially relevant to existing and emerging targeted therapies specifically in melanoma.

**Methods:**

The test utilizes the SNaPshot method (multiplex PCR, multiplex primer extension, and capillary electrophoresis) and can be performed rapidly with high sensitivity (requiring 5–10% mutant allele frequency) and minimal amounts of DNA (10–20 nanograms). The assay was validated using cell lines, fresh-frozen tissue, and formalin-fixed paraffin embedded tissue. Clinical characteristics and the impact on clinical trial enrollment were then assessed for the first 150 melanoma patients whose tumors were genotyped in the Vanderbilt molecular diagnostics lab.

**Results:**

Directing this test to a single disease, 90 of 150 (60%) melanomas from sites throughout the body harbored a mutation tested, including 57, 23, 6, 3, and 2 mutations in *BRAF*, *NRAS*, *GNAQ*, *KIT*, and *CTNNB1*, respectively. Among BRAF V600 mutations, 79%, 12%, 5%, and 4% were V600E, V600K, V600R, and V600M, respectively. 23 of 54 (43%) patients with mutation harboring metastatic disease were subsequently enrolled in genotype-driven trials.

**Conclusion:**

We present development of a simple mutational profiling screen for clinically relevant mutations in melanoma. Adoption of this genetically-informed approach to the treatment of melanoma has already had an impact on clinical trial enrollment and prioritization of therapy for patients with the disease.

## Introduction

Melanoma is a malignant tumor of melanocytes. Although the disease accounts for only 4% of all dermatologic cancers, it is responsible for 80% of deaths from skin cancer, with over 8,700 deaths projected in the USA in 2011 [1]. The 5 year survival for patients with metastatic disease not treated with surgical resection is well under 10%.

Historically, the disease has been classified based on histologic and morphologic findings of the tumor tissue as well as the anatomic site of origin. More recently, mutation profiling studies have revealed that melanoma is further comprised of clinically relevant molecular subsets defined by specific ‘driver’ mutations. Such mutations occur in genes that encode signaling proteins critical for cellular proliferation and survival. At least 6 genes have been shown to be recurrently mutated in melanoma, including the serine-threonine kinase encoded by *BRAF* [2]; the receptor tyrosine kinase encoded by *KIT* [3]; the GTP-binding proteins encoded by *NRAS* [4], *GNA11* [5], [6], and *GNAQ* [6], [7]; and the WNT signaling pathway component encoded by *CTNNB1* [8]. With the exception of *CTNNB1*, a tumor with an alteration in one of these genes rarely has a mutation in one of the other genes. Together, mutations in these genes can be found in approximately 70% of melanomas, depending on the site of origin of the primary lesion (**Table 1**). The frequency of gene mutation not only varies by site of origin but also by the presence or absence of chronic solar damage (CSD). For example, in skin intermittently exposed to sun, approximately 80% of melanomas have mutations in *BRAF* or *NRAS*. On the other hand, 15–20% of melanomas occurring on mucosal, acral, and CSD skin have a *KIT* mutation while few have *BRAF* mutations (5–15%). The KIT gene is wild-type (WT) in melanomas arising from skin without CSD [3].

**Table 1:** Gene mutation frequency in melanoma and predicted sensitivities to targeted.

Gene	Mutation	Frequency and Anatomic Site	Prediction
***BRAF***	V600E/R/K/M/G/D	8% CSD 58% non-CSD 22% Acral 3% Mucosal [2], [3], [4], [34]	Sensitive to: Vemurafenib GK2118436, GSK1120212 [9], [11], [33], [44], [45], [46], [47]
***NRAS***	G12C/S/R/V/A/D G13A/V/R/D Q61E/H/L/K/P/R	10% Acral 24% Mucosa 15% CSD 22% non-CSD [34], [48]	Sensitive to: (pre-clinical) MEK +/− PI3K inhibition [21], [49], [50], [51] MET inhibition [22] Resistant to: BRAF inhibitors
***KIT***	W557R V559A/D L576P K642E D816H	23% Acral 16% Mucosal 28% CSD [3], [39], [48]	Sensitive to: Imatinib Nilotinib Sunitinib Dasatinib Decreased sensitivity to: Imatinib (D816H only) [12], [16], [21], [39], [48]
***CTNNB1***	S37F/Y S45P/F/Y	4% Overall [52], [53], [54]	Preclinical progression of *BRAF*-mutant melanomas [8], [53]
***GNAQ***	Q209P/L/R	45% Uveal [6], [7]	Sensitive to: (pre-clinical) MEK inhibitors [55]

Tumor mutation status has been linked with sensitivity of melanomas to specific targeted therapies. Tumors that harbor BRAF V600E mutations display high radiographic response rates to mutant-specific inhibitors such as PLX4032/RG7204/vemurafinib (Plexxikon/Roche) [9], [10] and GSK2118436 (GlaxoSmithKline) [11], while patients whose tumors have certain *KIT* mutations (L576P, K642E, V559A) have disease sensitive to the KIT inhibitor, imatinib (**Table 1**) [12], [13], [14], [15], [16], [17], [18], [19], [20]. Preclinical data suggest that MEK inhibition with drugs like AZD6244 or GSK1120212 may be effective for uveal melanomas carrying *GNAQ* or *GNA11* mutations [6]. Tumors with *NRAS* mutations may respond to more potent MEK inhibitors (GSK1120212) or may require blockade of pathways mediated by both MEK and PI3K or other strategies directed at the MET receptor or ligand [21], [22].

We report here the development, validation, and clinical implementation of a multiplexed assay designed to simultaneously detect 43 recurrent mutations in *BRAF*, *KIT*, *NRAS*, *GNA11*, *GNAQ*, and *CTNNB1* using tumor-derived DNA from formalin-fixed paraffin-embedded (FFPE) tissues. The assay was adapted from a previously implemented genotyping platform designed for targeted mutational analysis of a broader set of tumor types [23]. The screen uses SNaPshot technology (Life Technologies/Applied Biosystems), which involves multiplexed amplification of DNA targets by the polymerase chain reaction (PCR) with unlabeled oligonucleotide primers, multiplexed single-base primer extension with fluorescently-labeled dideoxynucleotides, and analysis of labeled primer-extension products by capillary electrophoresis. Compared to direct sequencing, multiple publications have already documented that the SNaPshot assay offers higher analytical sensitivity and reduced complexity [23], [24], [25]. Our assay provides a robust and accessible approach for the rapid identification of important mutations in melanoma that can enable prioritization of specific targeted therapies. As proof of principle, we present our clinical experience with the initial 150 consecutive patients whose melanomas were prospectively screened and its impact on clinical trial assignment.

## Methods

### Cell Lines and Tumor Samples

Genomic DNA was derived from 16 cancer cell lines (10 melanoma cell lines and 6 additional various carcinoma cell lines used as additional positive and negative controls; **Table S7**) in addition to 24 fresh-frozen primary human melanomas. The following cancer cell lines were generously provided by Dr. David Solit (Memorial Sloan Kettering Cancer Center): WM1361A [26], SK-MEL-238 [27], SK-MEL-90 [28], MEL270 [29], and 92.1 [29]. The following cell lines were generously provided by Dr. Meenhard Herlyn (The Wistar Institute): WM1963 [30], WM3682 [30], WM115 [31], WM266-4 [2], and WM3211 [32]. The following cancer cell lines were available in the Pao laboratory: H358 [24], H2009 [24], H460 [24], H1975 [24], H1666 [24]. The LoVo [56] cell line is available commercially from the American Type Culture Collection (ATCC). DNA was either kindly provided by collaborators or was isolated using a DNeasy® kit (Qiagen Inc.). Some of these samples were subjected to whole genome amplification using the GenomiPhi DNA amplification kit (GE Healthcare) prior to use, as indicated. An additional 17 FFPE-derived DNA samples were extracted using a QIAamp DNA FFPE tissue kit (Qiagen, Inc.) and/or generously provided by collaborators. Human male genomic DNA (Promega Corporation) was used as a WT control.

### SNaPshot Assay

**Figure 1:**
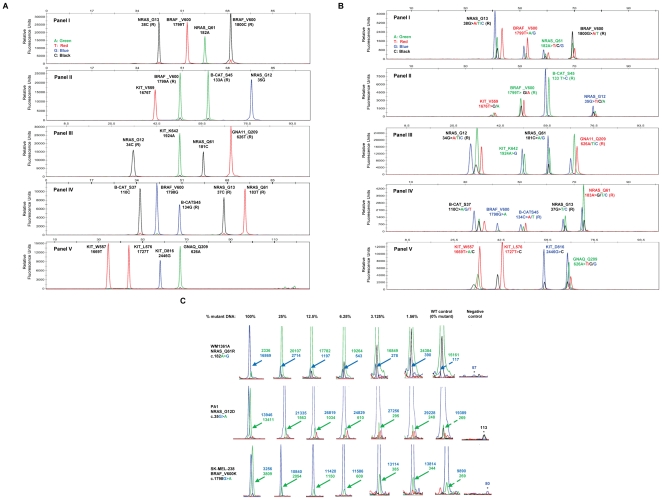
Melanoma SNaPshot screen (v1.0). A, five multiplexed panels can detect the mutational status of twenty gene loci. Each peak color represents a particular nucleotide at that locus. The gene name, amino acid, and nucleotide are labeled above each peak. An “(R)” after the nucleotide denotes a reverse extension primer. B, pan-positive control for melanoma SNaPshot screen. Peaks are labeled as described in A. C, SNaPshot sensitivity measurement using cell line DNA carrying known mutations. Numbers indicate the arbitrary fluorescence units of WT (panel 1: green, panels 2, 3: blue) and mutant (panel 1: blue, panels 2, 3: green) peaks. Solid arrows indicate mutant peaks and dotted arrows show background peaks. Background peaks in the negative controls (far right panel) are indicated by their peak height and a star (*).

The basic SNaPshot technique for cancer mutation analysis has been described [23]. The standard operating procedure protocol is provided in the **Methods S1**. PCR primers for this specific melanoma screen are listed in **Table S1**. Single-base extension primers are listed in **Table S2**. The concentration of PCR and extension primers in each panel were optimized so that all fluorescently labeled fragments displayed similar peak heights after capillary electrophoresis (**Figure 1A**). Each peak was individually validated with DNA from cell lines containing known mutations or ‘spiking primers’ (i.e. oligonucleotides; **Table S3**) harboring mutations of interest (**Figure S1A–E**). For each panel, the spiking primers were mixed to create a pan-positive control mix using pools of ‘spiking primers’ (**Table S4**) to detect all possible known mutations at each site (**Figure 1B**). Using genomic DNA from frozen tissue samples, we were able to reliably perform the entire SNaPshot screen with all five panels using 20 nanograms of DNA per panel.

To measure assay sensitivity, one representative mutation in each of the five panels was studied, using mixtures of male human non-neoplastic genomic DNA and DNA from positive control cell lines with known mutations. Cell lines SK-MEL-238, PA1, and WM1361A were used as examples for sensitivity measurements of BRAF V600K, NRAS G12D, and NRAS Q61R mutations, respectively (**Figure 1C** and data not shown). For any given locus, a mutation was called confidently if its peak height was greater than or equal to 10% of the corresponding heterozygous WT peak in the same sample. If the height of a potential mutation peak was less than 10% of the corresponding WT peak or if no WT peak was detected, then a mutation was called if the potential mutant peak was three times higher than any background peaks of the same color and size in separate analyses of WT DNA controls [24]. The y-axis was adjusted to the appropriate scale to visualize various peaks. According to these criteria, mutant peaks were visually observed in dilutions as low as 6.25%, consistent with prior published results on two different SNaPshot screens [23], [24].

The current screen was designed to distinguish between *BRAF* mutations in *cis* or *trans*. If a two nucleotide *BRAF* mutation (e.g. 1798_1799GT>AA) is present in *cis*, the 1799 mutation will not be detected in the forward direction (Panel I), but will be detected using the 1799 reverse primer (Panel 2). If the two nucleotide *BRAF* mutation is present in *trans*, the 1799 mutation should be detected using both the forward and reverse primers (**Table S2**).

### Direct Dideoxynucleotide-Based Sequencing

All test and validation samples with mutations detected by SNaPshot underwent secondary analysis by direct sequencing as published using M13-tagged gene-specific primers (**Table S5**) [24].

### Assessment of Clinical Tumor Samples

The first consecutive 150 melanoma samples in the molecular diagnostics lab and associated clinical characteristics were analyzed after obtaining written informed consent from all patients on a Vanderbilt University Institutional Review Board (IRB) - approved protocol (MEL #09109). All clinical data was obtained and maintained according to HIPAA standards. All unique identifiers have been removed prior to publication. Tissue and tumor samples in this study were obtained from Vanderbilt University and were all used under the Vanderbilt University Institutional Review Board (IRB) - approved protocol IRB# 100178 entitled “VICC MEL 09109-Storage and Research Use of Human Biospecimens from Melanoma Patients and Clinical Testing for the Assignment of Therapy”.

## Results

### Development of a SNaPshot Assay to Assess Multiple Somatic Point Mutations in Melanoma

The melanoma SNaPshot screen (v1.0) interrogates 43 somatic point mutations occurring at 20 different loci in 6 genes (**Table 2**). These mutations were originally selected in 2009 because they: 1) appear in melanomas, 2) could potentially be used to prioritize selection of existing or emerging targeted therapy, and 3) occur at mutational ‘hotspots’. The screen included 21 single-base extension SNaPshot assays, a portion of which were derived from a 58 mutation genotyping panel that is currently being used for clinical testing of FFPE-derived tumor samples [23]. While other genes (e.g. *CDKN2C*, *CDKN2A*, *MITF*, *BAP1*, *PTEN*, *ERBB4*, and *FGFR2*, etc.) are mutated with some frequency in melanoma, no common recurring mutations in these genes are observed or the function of observed mutations are unknown; therefore these genes were not included in our screen. The selected mutations were incorporated into five multiplexed panels, each capable of detecting mutations at four (Panels I, II, III and V) or five (Panel IV) loci (**Figure 1A**).

**Table 2:** The SNaPshot melanoma screen can detect 43 point mutations in 6 genes relevant to targeted therapy in melanoma.

*NRAS*			*KIT*		
Position	AA mutant	Nucleotide mutant*	Position	AA mutant	Nucleotide mutant
**G12**	p.G12C	**c.34G>T**	**W557**	p.W557R	c.1669T>C
	p.G12S	**c.34G>A**		p.W557R	c.1669T>A
	p.G12R	c.34G>C	**V559**	p.V559A	c.1676T>C
	p.G12V	**c.35G>T**		p.V559D	c.1676T>A
	p.G12A	**c.35G>C**	**L576**	p.L576P	c.1727T>C
	p.G12D	**c.35G>A**	**K642**	p.K642E	c.1924A>G
**G13**	p.G13A	c.38G>C	**D816**	p.D816H	c.2446G>C
	p.G13V	**c.38G>T**			
	p.G13R	**c.37G>C**	***CTNNB1***		
	p.G13D	**c.38G>A**	**S37**	p.S37F	**c.110C>T**
**Q61**	p.Q61E	c.181C>G		p.S37Y	**c.110C>A**
	p.Q61H	**c.183A>T**	**S45**	p.S45P	**c.133T>C**
	p.Q61H	**c.183A>C**		p.S45F	**c.134T>C**
	p.Q61L	**c.182A>T**		p.S45Y	c.134C>A
	p.Q61L	c.182_183AA>TG			
	p.Q61K	**c.181C>A**	***GNA11***		
	p.Q61P	**c.182A>C**	**Q209**	p.Q209P	c.626A>C
	p.Q61R	**c.182A>G**		p.Q209L	c.626A>T
	p.Q61R	c.182_183AA>GG			
			***GNAQ***		
***BRAF***			**Q209**	p.Q209P	c.626A>C
**V600**	p.V600R	c.1798_1799GT>AG		p.Q209L	c.626A>T
	p.V600K	c.1798_1799GT>AA		p.Q209R	c.626A>G
	p.V600E	**c.1799T>A**			
	p.V600E	c.1799_1800TG>AA			
	p.V600M	**c.1798G>A**			
	p.V600G	c.1799T>G			
	p.V600D	c.1799_1800TG>AT			

* SNaPshot assays in bold text were previously published [23].

### Distinguishing Among Different Mutant BRAF Alleles at Amino Acid V600

According to the Catalogue of Somatic Mutations in Cancer (COSMIC), approximately 42% of melanomas harbor *BRAF* mutations, of which 36% are V600E and 3% are V600R/K/M/G/D. Although mutant-specific inhibitors like vemurafenib and GSK2118436 are predicted to be equally efficacious against a variety of V600 mutants [33], clinical trials with the approved BRAF inhibitor, Vemurafenib, have thus far have focused on enrolling only those with V600E mutant melanoma. Therefore, we designed our SNaPshot platform to distinguish among BRAF V600 mutants (**Table 2**). DNA from fresh-frozen or FFPE human melanoma tissue was used to show detection of multiple BRAF V600 mutations V600E/K/M/R/E (**Figure S2**).

### Validation of the Melanoma SNaPshot Screen on Tumor Samples

We used the SNaPshot screen to interrogate a panel of 16 cell lines with known mutation status. Results were in 100% concordance with previously published data; no false positive or false negative cases were observed (**Table S6** and **Table S7**). The lack of detection of mutations in known WT samples or samples with mutations in homologous genes (e.g. *NRAS* vs. *KRAS*) demonstrates specificity of the SNaPshot assay.

We next interrogated the mutation status of 24 fresh-frozen primary human melanomas using the SNaPshot screen (**Figure S3**, **Table S6** and **S8**). Thirteen tumors (54%) had BRAF mutations including 10 V600Es, 2 V600Ks, and 1 V600M. Three samples (12.5%) had NRAS mutations: 2 Q61Rs and 1 G13A. One sample (4.2%) had a KIT L567P mutation. The remaining samples were WT for all of the mutations tested. As expected, *BRAF*, *NRAS*, and *KIT* mutations were mutually exclusive, and the distribution of mutations was consistent with that reported in the literature (**Table 1**) [2], [3], [4], [34]. All mutations detected by the SNaPshot assay were verified by direct sequencing.

Finally to complete the development phase, the assay was used to evaluate DNA from 18 FFPE samples (**Table S6 and S9**). Seven samples (OHSU10) had known mutational status and were evaluated blinded. The other 11 samples (VICC) had previously unknown mutational status. Six samples harbored KIT mutations, including 2 W557Rs, a V559A, a V559R, a L576P, and a K642E. Seven samples contained BRAF mutations, including 4 V600Es, 2 V600Ks, and 1 V600R. One sample had an NRAS G13D mutation, and four samples were WT for all mutations tested. A tumor with a mutation in one gene did not harbor a mutation in any other gene. We achieved 100% concordance with known results.

### Spectrum of Mutations in the First 150 Clinically Screened Melanomas

**Figure 2:**
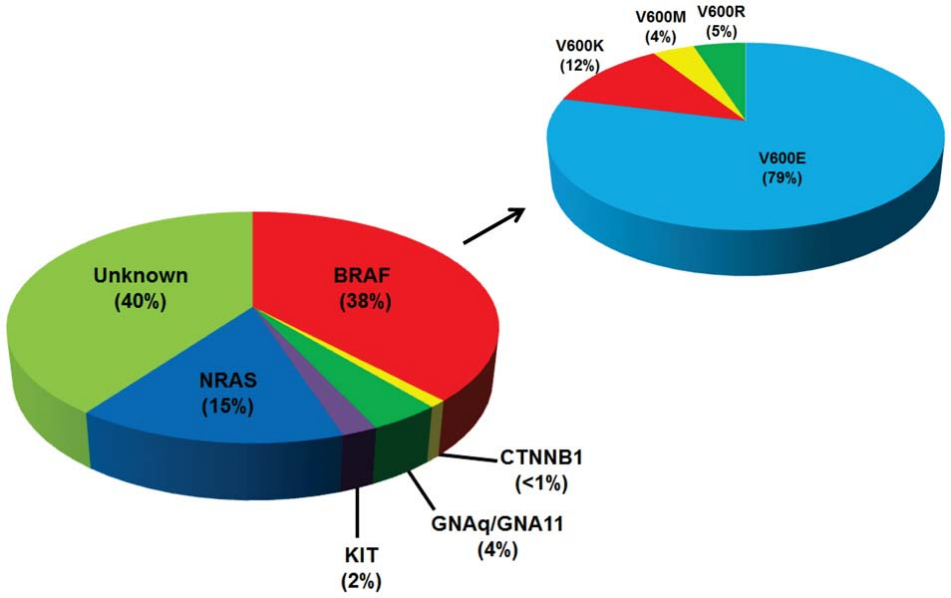
Distribution of mutations in the first 150 tumors genotyped in the molecular diagnostic lab. Left: distribution of all mutations. Right: distribution of V600 mutations. See **Table S10** for more details.

In July 2010, the SNaPshot assay was implemented in Vanderbilt's Clinical Laboratory Improvement Amendments-approved Molecular Diagnostics Laboratory as a component of routine care for patients with melanoma. Among the first 150 melanomas genotyped with informed consent (from 07/08/2010 to 12/13/2010), 90 (60%) had at least one mutation (**Table 3**, **Figure 2**; **Table S10**), including 57, 23, 6, 3, and 2 mutations in *BRAF*, *NRAS*, *GNAQ*, *KIT*, and *CTTNB1*, respectively. Among BRAF V600 mutations, 79%, 12%, 5%, and 4% were V600E, V600K, V600R, and V600M, respectively. Among the 57 melanomas with BRAF V600 mutations, 35 originated from intermittent sun damaged skin, 10 from chronic sun damaged skin, 2 from acral sites, 2 from mucosal sites, and 8 from unknown primary sites. None of the 7 uveal melanomas contained *BRAF* mutations. *NRAS* mutations were found in disease from all sites except the uvea. 2 of 3 *KIT* changes were found in melanomas from acral and mucosal primary sites. 5 of 6 *GNAQ* mutations were found in melanomas from uveal sites. No mutations were found in *GNA11* in this small set of uveal melanomas. Only one tumor had two mutations (NRAS Q61L and CTNNB1 45P), while all other mutations were mutually exclusive.

**Table 3:** Spectrum of mutations in the first 150 melanomas genotyped in the molecular diagnostic lab.

Site of primary	# of cases	Mutation Present	*BRAF*	*CTNNB1*	*GNAQ/GNA11*	*KIT*	*NRAS*	No mutation detected
Head and Neck (CSD*)	27	11 (41%)	10	0	0/0	0	1	16
Torso (non-CSD)	46	32 (70%)	24	1#	0/0	1	7	14
Extremities (non-CSD)	31	19 (61%)	11	0	0/0	0	8	12
Uveal	7	5 (71%)	0	0	5/0	0	0	2
Acral	13	6 (46%)	2	1	0/0	1	2	7
Mucosal	11	5 (45%)	2	0	0/0	1	2	6
Unknown primary	15	12 (80%)	8	0	1/0	0	3	3
Total cases (% of total)	150	90 (60%)	57 (38%)	2 (1.4%)	6 (4%)	3 (2%)	23 (15%)	60 (40%)

* CSD – chronic sun damage.

# This CTNNB1 mutation (CTNNB1 S45P) occurred concurrently with an NRAS Q61L mutation.

### Clinical Trial enrollment of metastatic melanoma patients with detected mutations

Eighty-two patients had metastatic (M1) disease of which 54 had mutations. The prospective nature of this study provides a better understanding on the impact of the implementation of the test and its effect on patient treatment selection. Importantly, 23 of 54 patients (43%) with metastatic disease containing a detectable mutation were subsequently enrolled on genotype-driven trials (**Table 4**). This is not restricted only to patients with BRAF V600E. Patient with NRAS, KIT, and GNAQ mutations were also enrolled on specific trials directed at their tumor mutation status. These data demonstrate the utility of this approach to the treatment of melanoma and its ability to better match patients with more effective therapies.

**Table 4:** Influence of tumor genotype on subsequent treatment in patients with metastatic melanoma.

Gene	# of metastatic cases	# patients placed on a genotype- driven clinical trial (%)
*BRAF*	32	14 (44%)
*CTNNB1*	1*	1 (100%)
*GNAQ/GNA11*	6	3 (100%)
*KIT*	1	1 (100%)
*NRAS*	15	5 (33%)
No mutation detected	28	N/A
**Total cases**	**82**	**23/54 (43%)**

* This CTNNB1 mutation (CTNNB1 S45P) occurred concurrently with an NRAS Q61L mutation.

## Discussion

Historically, therapeutic decisions for the treatment of malignant melanoma have been based upon stage and histology (ulceration and depth or volume of tumor), with the choice of systemic anti-cancer therapies guided mostly by empiric data leading to generally dismal outcomes [35], [36], [37], [38]. However, basic and translational research has uncovered molecular abnormalities in melanomas that not only drive and sustain the cancer but can also serve as attractive therapeutic targets. For example, mutant-specific inhibitors induce a >50% response rate in patients with BRAF V600-mutant tumors [9], [10], [11], [39], [40], and nearly 50% of tumors harboring certain *KIT* mutations are highly sensitive to imatinib [12], [15], [16], [18], [19], [20].

Here, we present development, validation, and clinical implementation of a disease-specific SNaPshot-based screen [23], [24] to assess melanoma tumor samples simultaneously for 43 somatic recurrent point mutations in 6 genes with relevance to targeted therapy. The SNaPshot assay can be performed rapidly with minimal amounts of starting FFPE-derived DNA material (20 nanograms) and high sensitivity [23], [24], detecting mutations in samples when mutant DNA comprises <10% of the total DNA (see supplemental material). By comparison, direct dideoxynucleotide sequencing, used currently in many clinical molecular labs, requires that mutant DNA comprise >20–25% of the total DNA for mutation detection.

In its present form, the SNaPshot assay can detect mutations that occur in the majority of melanomas. Of the first 150 tumor samples tested in the clinical lab, 90 (60%) had an identifiable mutation, which were 38% *BRAF*, 15% *NRAS*, 4% *GNAQ*, 2% *KIT*, and ∼1% *CTNNB1*. Now with additional prospective testing for over 15 months, the numbers remain similar in their breakdown. The frequency of these mutations and the anatomic sites of origin for the primary tumor were consistent with previously published results. Of the 90 mutations detected, 57 mutations were identified that involved the BRAF V600 position. The percent of BRAF mutations that were V600E (79%) (**Figure 2**) is also consistent with what has been reported in the literature [2], [34], [41]. Since our assay was designed to distinguish among various mutations that affect V600, our data further show that allele-specific molecular diagnostic assays designed to detect only the most common V600E mutation will miss ∼20% of the total number of V600 mutations in melanoma.

Importantly, our results demonstrate the impact of tumor mutation assessment on directing melanoma patients to the most appropriate clinical trials with the therapeutic agents most likely to provide a benefit. Of the 54 patients with metastatic disease and a detected tumor mutation, 23 (43%) were subsequently enrolled onto genotype-driven trials based upon the results from their tumor mutational profiling. This is a dramatic advantage over a simple allele specific PCR for BRAF V600E. In addition to BRAF inhibitors, patients are directed to trials for KIT mutations, GNAQ/11 mutations in uveal melanoma, and even NRAS mutant melanoma. In addition, studies in patients who have disease progression following initial response to BRAF inhibitor therapy have revealed a secondary mutation in NRAS as the mechanism of resistance in nearly a quarter of this patient population [42]. Therefore, mutational profiling of resistant disease after BRAF inhibitors may provide insight into selecting secondary therapy.

This prospective approach to mutation analysis has multiple advantages in melanoma. First and foremost, it allows prospective patient selection to the best available therapies or most relevant clinical trials based on tumor mutational status. Given the increasing number of clinically relevant genotypes in melanoma and the expanding repertoire of targeted inhibitors (**Table S11**), clinical characteristics or tumor histology are no longer the most effective way to select and prioritize treatment options for patients with this disease. A single comprehensive tumor genotyping panel in the form of the SNaPshot test will allow patients and physicians to understand and incorporate complex tumor-gene-mutation information into their treatment algorithms. Second, because the disease can quickly progress, determining tumor mutation status as part of routine care enables faster treatment prioritization [43]. Third, prospective genotyping allows for the determination of an accurate portrait of the genomic profile of patients who are routinely referred to this institution as opposed to retrospective studies reported from large databases (e.g. COSMIC) or other institutions. Finally, prospective tumor profiling may allow us to make previously unknown associations between a tumor mutation and clinical features and/or clinical activity of new drug combinations. Of greatest importance, this assay has proven benefit in directing patients to the most appropriate therapies and clinical trials, which will ultimately lead to improved outcomes for patients with melanoma.

## Supporting Information

Figure S1 **The melanoma screen can detect various BRAF V600 mutations.** BRAF V600 status and the *BRAF* nucleotide(s) detected by SNaPshot are indicated to the left of the panels. The SNaPshot panels that detect the *BRAF* nucleotides are specified above the peaks. Forward extension primers are represented by ‘F’ and reverse extension primers are represented by ‘R’. Representative *BRAF* mutations are shown: A, WT BRAF V600, B, BRAF V600E, C, BRAF V600E, D, BRAF V600K, E, BRAF V600M, and F, BRAF V600R.
(TIF)

Figure S2 **Validation of each SNaPshot peak.** DNA from FFPE samples with known mutation status or spiking primers containing mutations of interest were used to validate the detection of each mutation in the screen. Validation of mutations was performed as described in the Materials and Methods section (A) Panel I, (B) Panel II, (C) Panel III, (D) Panel IV, and (E) Panel V.
(TIF)

Figure S3 **Melanoma SNaPshot screen results confirmed by direct sequencing.** DNA from frozen melanoma samples (see **Table S8**) was extracted and subject to the melanoma SNaPshot assay (left panels) and direct sequencing (right panels). The arrows indicate the position of the mutated peaks. Representative samples with mutations in *BRAF*, *NRAS*, and *KIT* are shown. All traces are available upon request.
(TIF)

Methods S1 **Standard operating procedure: SNaPshot genotyping assay for melanoma.**
(DOCX)

Table S1 **PCR primers for SNaPshot screen.**
(DOC)

Table S2 **Single-base extension primers for SNaPshot screen.**
(DOC)

Table S3 **Spiking primers used for pan-positive control assay.**
(DOC)

Table S4 **Pan-positive control mix preparation.**
(DOC)

Table S5 **PCR primers used for direct sequencing.**
(DOC)

Table S6 **Summary of mutations detected in cell lines, frozen tissues, and FFPE samples.**
(DOC)

Table S7 **SNaPshot assay results for cell lines.**
(DOC)

Table S8 **SNaPshot assay results for fresh-frozen primary human melanomas.**
(DOC)

Table S9 **SNaPshot assay results for FFPE tissue.**
(DOC)

Table S10 **SNaPshot assay results for the first 150 clinically screened melanomas.**
(DOCX)

Table S11 **Open genotype-driven clinical trials at Vanderbilt University.**
(DOC)
