# Supplementary figures and images for: Routine Multiplex Mutational Profiling of Melanomas Enables Enrollment in Genotype-Driven Therapeutic Trials
Source: PLoS One. 2012 Apr 20;7(4):e35309. doi: 10.1371/journal.pone.0035309 (PMC3335021; doi:10.1371/journal.pone.0035309)

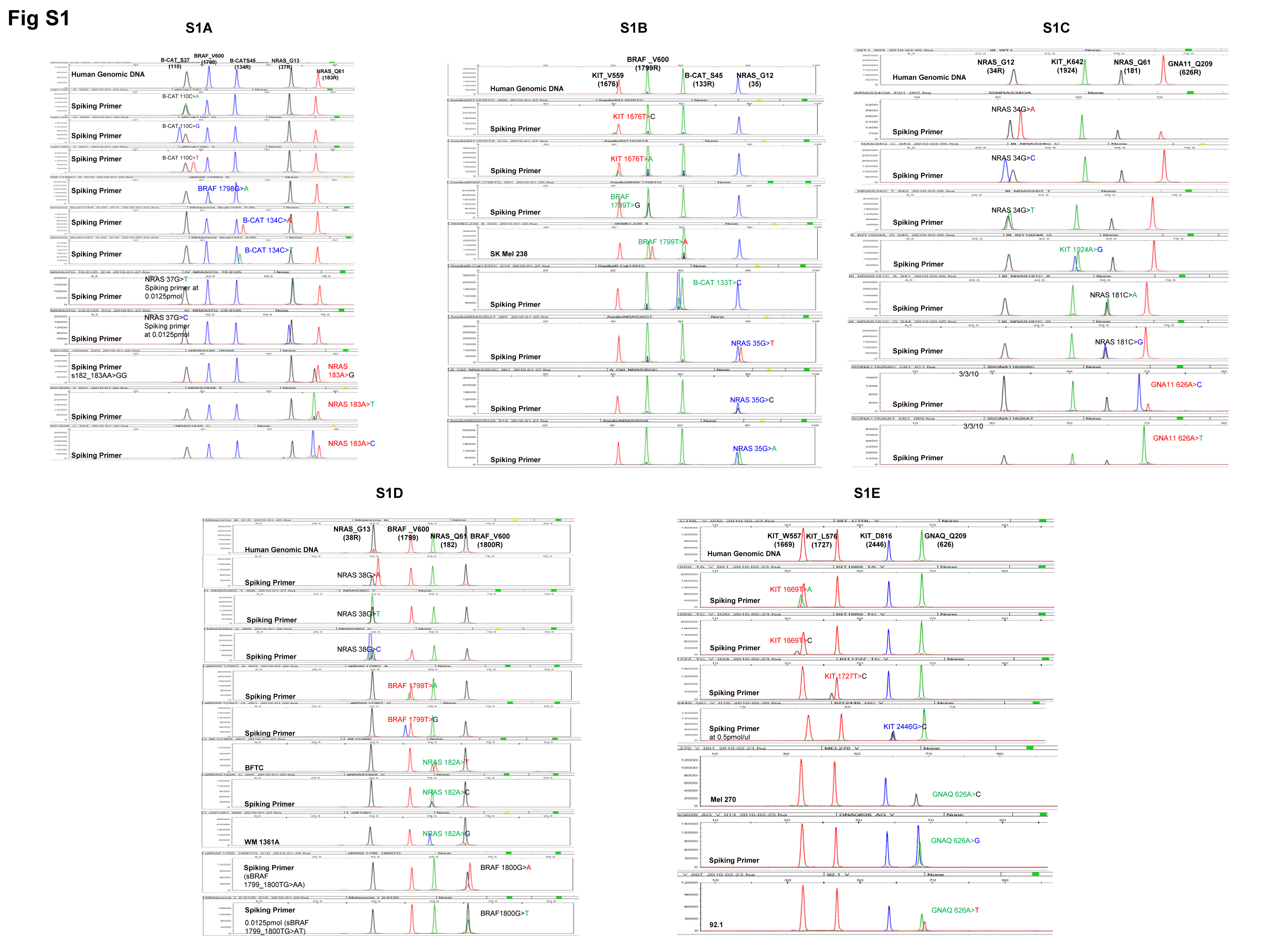

Supplement: Figure S1 — The melanoma screen can detect various BRAF V600 mutations. BRAF V600 status and the BRAF nucleotide(s) detected by SNaPshot are indicated to the left of the panels. The SNaPshot panels that detect the BRAF nucleotides are specified above the peaks. Forward extension primers are represented by ‘F’ and reverse extension primers are represented by ‘R’. Representative BRAF mutations are shown: A, WT BRAF V600, B, BRAF V600E, C, BRAF V600E, D, BRAF V600K, E, BRAF V600M, and F, BRAF V600R. (TIF) [file pone.0035309.s001.tif]

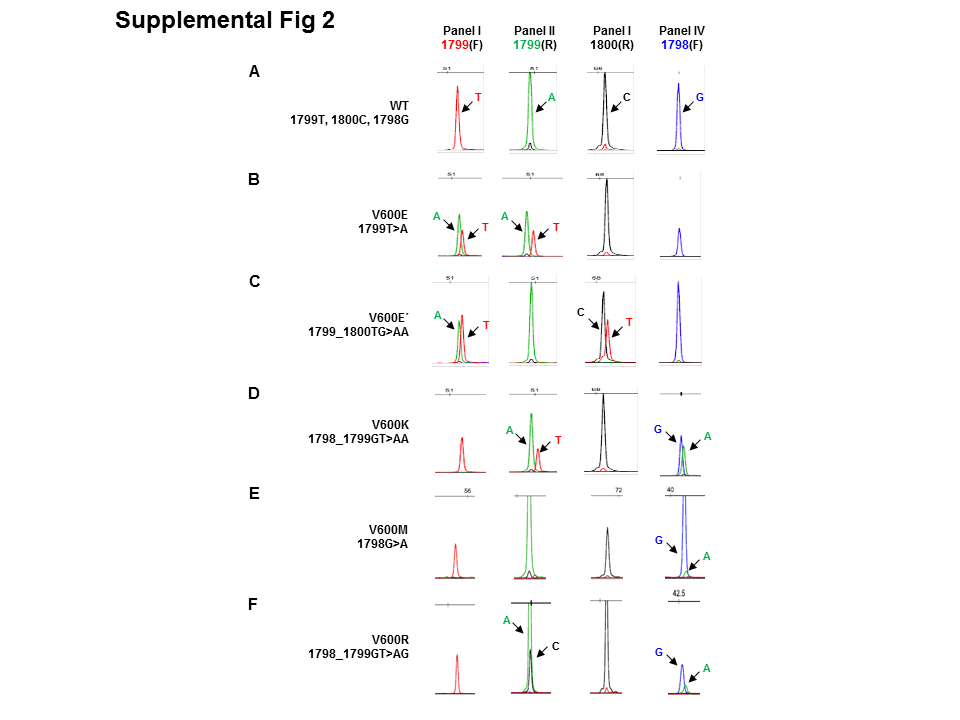

Supplement: Figure S2 — Validation of each SNaPshot peak. DNA from FFPE samples with known mutation status or spiking primers containing mutations of interest were used to validate the detection of each mutation in the screen. Validation of mutations was performed as described in the Materials and Methods section (A) Panel I, (B) Panel II, (C) Panel III, (D) Panel IV, and (E) Panel V. (TIF) [file pone.0035309.s002.tif]

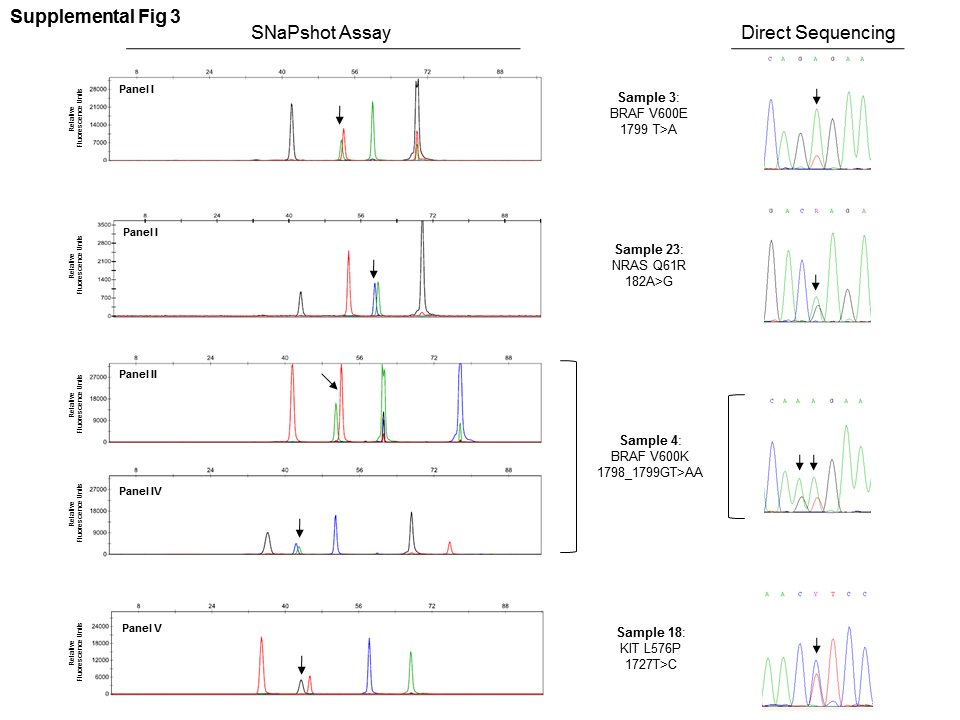

Supplement: Figure S3 — Melanoma SNaPshot screen results confirmed by direct sequencing. DNA from frozen melanoma samples (see Table S8) was extracted and subject to the melanoma SNaPshot assay (left panels) and direct sequencing (right panels). The arrows indicate the position of the mutated peaks. Representative samples with mutations in BRAF, NRAS, and KIT are shown. All traces are available upon request. (TIF) [file pone.0035309.s003.tif]
