# Supplementary material for: Routine Multiplex Mutational Profiling of Melanomas Enables Enrollment in Genotype-Driven Therapeutic Trials
Source: PLoS One. 2012 Apr 20;7(4):e35309. doi: 10.1371/journal.pone.0035309 (PMC3335021; doi:10.1371/journal.pone.0035309)
Supplement: Methods S1 — Standard operating procedure: SNaPshot genotyping assay for melanoma. (DOCX) [file pone.0035309.s004.docx]

**Methods S1**

**Standard operating procedure: SNaPshot genotyping assay for melanoma**

**Table of Contents**

**Sections Pages**

**Background ………………………………………………………… 3**

**SNaPShot Genotyping Working Area ……………………………. 4**

**Materials and Equipment ………………………………………….. 4**

**Reagents …………………………………………………………….. 5**

**Multiplex PCR & Extension Primers…………………………….... 6-7**

**Primer Solution Preparation ……………………………………..... 8-12**

**SNaPShot Genotyping Procedures ……………………………….... 13-18**

**Appendices ……………………………………………………………. 21-23**

**References ……………………………………………………………. 24**

**Background**

Signaling pathways play key roles in the regulation of cell death and proliferation and genetic alterations in the signaling molecules can result in cancers. Drugs targeting key signaling molecules have been successfully used for treating cancer patients. Importantly, the sensitivity of these drugs is highly related to the genetic makeup of individual tumors. Thus, mutational profiles of tumors can be used to prioritize anti-cancer therapy. Recently, investigators at Massachusetts General Hospital (MGH) have developed a fast and high-throughput multiplex mutational profiling method based on Applied Biosystems ‘SNaPshot’ platform involving multiplex PCR, primer extension, and capillary electrophoresis. Mutations are detected when mutant DNA comprises < 10% of the total DNA. ~10-20 ng of DNA is used per panel. Based on mutational databases and published reports, we have developed a specific screen for melanoma that assesses 43 somatic mutations in 6 genes (*BRAF*, *NRAS*, *KIT*, *GNAQ*, *GNA11*, and *CTNNB1*) (Table 1).

**Table 1.** The SNaPshot melanoma screen can detect 43 point mutations in 6 genes relevant to targeted therapy in melanoma.

| ***NRAS*** |  |  |  | ***KIT*** |  |  |
| --- | --- | --- | --- | --- | --- | --- |
| **Position** | **AA mutant** | **Nucleotide mutant^*^** |  | **Position** | **AA mutant** | **Nucleotide mutant** |
| G12 | p.G12C | **c.34G>T** |  | W557 | p.W557R | c.1669T>C |
|  | p.G12S | **c.34G>A** |  |  |  | c.1669T>A |
|  | p.G12R | c.34G>C |  | V559 | p.V559A | c.1676T>C |
|  | p.G12V | **c.35G>T** |  |  | p.V559D | c.1676T>A |
|  | p.G12A | **c.35G>C** |  | L576 | p.L576P | c.1727T>C |
|  | p.G12D | **c.35G>A** |  | K642 | p.K642E | c.1924A>G |
| G13 | p.G13A | c.38G>C |  | D816 | p.D816H | c.2446G>C |
|  | p.G13V | **c.38G>T** |  |  |  |  |
|  | p.G13R | **c.37G>C** |  | ***CTNNB1*** |  |  |
|  | p.G13D | **c.38G>A** |  | S37 | p.S37F | **c.110C>T** |
| Q61 | p.Q61E | c.181C>G |  |  | p.S37Y | **c.110C>A** |
|  | p.Q61H | **c.183A>T** |  | S45 | p.S45P | **c.133T>C** |
|  |  | **c.183A>C** |  |  | p.S45F | **c.134T>C** |
|  | p.Q61L | **c.182A>T** |  |  | p.S45Y | c.134C>A |
|  |  | c.182_183AA>TG |  |  |  |  |
|  | p.Q61K | **c.181C>A** |  | ***GNA11*** |  |  |
|  | p.Q61P | **c.182A>C** |  | Q209 | p.Q209P | c.626A>C |
|  | p.Q61R | **c.182A>G** |  |  | p.Q209L | c.626A>T |
|  |  | c.182_183AA>GG |  |  |  |  |
|  |  |  |  | ***GNAQ*** |  |  |
| ***BRAF*** |  |  |  | Q209 | p.Q209P | c.626A>C |
| V600 | p.V600R | c.1798_1799GT>AG |  |  | p.Q209L | c.626A>T |
|  | p.V600K | c.1798_1799GT>AA |  |  | p.Q209R | c.626A>G |
|  | p.V600E | **c.1799T>A** |  |  |  |  |
|  |  | c.1799_1800TG>AA |  |  |  |  |
|  | p.V600M | **c.1798G>A** |  |  |  |  |
|  | p.V600G | c.1799T>G |  |  |  |  |
|  | p.V600D | c.1799_1800TG>AT |  |  |  |  |

^*^SNaPshot assays in bold text were previously published ([1](#_ENREF_1)).

**SNaPShot Genotyping Working Area**

**Area 1:** Pre-PCR preparation area. Contains a UV-lamp PCR hood, a vortex mixer, a spinner, a -20 ºC freezer and a 4 ºC refrigerator. Area 1 should be completely DNA free and separated from any DNA sources. No patient sample DNA and amplified DNA are permitted in this area. All the PCR and extension primers should be prepared here in the PCR hood.

**Area 2:** Specimen preparation area. Contains a -20 ºC freezer, a vortex mixer and a spinner. DNA extraction is performed in this area. In addition, the extracted sample DNA is added into PCR reaction tubes in this area.

**Area 3:** PCR amplification and post PCR area. Contains a PCR cycler, which is designated only for SNaPShot use, a -20 ºC freezer, a 4 ºC refrigerator, a vortex mixer, a regular spinner and a 96-well plate spinner. PCR and extension reactions are performed in this area. In addition, loading samples for the ABI 3730 are prepared in this area.

***Notes:***

1. *Work under nuclease-free conditions.*
2. *Wear gloves at all times, and change gloves frequently.*
3. *Clean up all work surfaces (including bench areas adjacent to the PCR hood) with bleach, ethanol and RNase Away.*
4. *Always work in one direction, moving from Area 1 to Area 2 to Area 3. Any DNA or amplified PCR products should NEVER be returned to Area 1.*

**Materials and Equipment**

1. 0.2 ml PCR 8-tube strips with lids
2. 1.5 ml microcentrifuge tubes
3. 10 µL filter tips
4. 20 µL filter tips
5. 200 µL filter tips
6. 1000 µL filter tips
7. Adhesive PCR film
8. MicroAmp® Optical 96-well reaction plate; ABI (#N801-0560)
9. 96-well plate septa; ABI (#4315933)
10. 0.5-10 µL pipet
11. 2-20 µL pipet
12. 10-200 µL pipet100-1000 µL pipet
13. Micro-centrifuge
14. Spinner
15. Vortex mixer
16. PCR cycler
17. Applied Biosystems 3730 DNA Analyzer

**Reagents**

*The following reagents are stored in Area 1

1. UltraPure^TM^ Distilled Water (DNase, RNase free). GIBCO. Store at room temperature
2. 10 mM dNTP Mix. Invitrogen. Store at -20 ºC.
3. dNTP working solution (2 mM) preparation. Add 4 mL of nuclease-free water into the 1 mL dNTP stock (10 mM). Mix thoroughly by vortexing. Make 200 µL aliquots and store the tubes at –20 ºC for use.
4. Platinum Taq (5 units/µL). Invitrogen. Store at -20 ºC.
5. ABI PRISM® SNaPShot® Multiplex Kit. ABI. Store at -20 ºC.
6. SNaPShot RR Mix working solution preparation. Add 700 µL of nuclease-free water into 500 µL of RR mix located in ‘SNaPshot® Multiplex Kit’. Mix thoroughly by vortexing, make 600 µL aliquots and store the tubes at -20 ºC for use.

* The following reagent is stored in Area 2

1. Human Genomic DNA (male). Promega (#G147A). Store at -20 ºC. Dilute to 5 ng/µL for use.

* The following reagents are stored in Area 3

1. Exonuclease I (Exo I). USB. Store at -20 ºC.
2. Shrimp Alkaline Phosphatase (SAP). USB. Store at -20 ºC.
3. Hi-Di Formamide. ABI (#4311320). Store at -20 ºC.
4. GeneScan^TM^120 LIZ^TM^ Size Standard. ABI (#4324287). Store at 4 ºC.
5. POP-7 Polymer. ABI (#4363929). Store at 4 ºC.

**Multiplex PCR & extension Primers**

***Upon receipt, store the following primers at 4 ºC in Area 1***

**Table 2. Multiplex PCR Primers**

| **Amplification primer name** | **Primer sequence^a^** | **Length of product (bp)** |
| --- | --- | --- |
| BRAF_ex15_a1^b^ | ACGTTGGATGTGCTTGCTCTGATAGGAAAATG | 143 |
| BRAF_ex15_a2^b^ | ACGTTGGATGCTGATGGGACCCACTCCAT |  |
| B-Catenin_ex3_a1^b^ | ACGTTGGATGTCACTGGCAGCAACAGTCTT | 89 |
| B-Catenin_ex3_a2^b^ | ACGTTGGATGCAGGATTGCCTTTACCACTCA |  |
| GNA11ex5F | TGCAGATTGGGCCTTGGGGC | 197 |
| GNA11ex5R | GCAGGGCCCACCTCGTTGTC |  |
| GNAQex5A^c^ | CCCACACCCTACTTTCTATCATTTAC | 298 |
| GNAQex5B^c^ | TTTTCCCTAAGTTTGTAAGTAGTGC |  |
| KIT642F | GCGGCCATGACTGTCGCTGT | 251 |
| KIT642R | AGGCAGCTTGGACACGGCTT |  |
| KIT557-576F | TCTCCAGAGTGCTCTAATGACTGAGAC | 189 |
| KIT557-576R | GCCTGTTTCTGGGAAACTCCCATT |  |
| KIT_ex17_a1^b^ | ACGTTGGATGTCATGGTCGGATCACAAAGA | 98 |
| KIT_ex17_a2^b^ | ACGTTGGATGGAGAATGGGTACTCACGTTTCC |  |
| NRAS_ex2_a1^b^ | ACGTTGGATGCAACAGGTTCTTGCTGGTGT | 175 |
| NRAS_ex2_a2^b^ | ACGTTGGATGGAGAGACAGGATCAGGTCAGC |  |
| NRAS_ex3_a1^b^ | ACGTTGGATGTGGTGAAACCTGTTTGTTGG | 179 |
| NRAS_ex3_a2^b^ | ACGTTGGATGCCTTTCAGAGAAAATAATGCTCCT |  |

^a^The sequences are shown 5’>3’.

^b^Primer sequences were published previously.([1](#_ENREF_1))

^c^Primer sequences were published previously.([2](#_ENREF_2))

**Table 3. Single-base extension primers.**

| **Extension primer**  **name^a^** | **Primer sequence^b^** | **Primer length (nucleotides)** |
| --- | --- | --- |
| BRAF1798_extF | **CTGACTGACTGACT**GGTGATTTTGGTCTAGCTACA | 35 |
| BRAF1799_extF^c^ | **GACTGACTGACTGACTGACTGACT**GTGATTTTGGTCTAGCTACAG | 45 |
| BRAF1799_extR | **ACTGACTGACTGACTGACTGACTGACTG**CACTCCATCGAGATTTC | 45 |
| BRAF1800_extR | **GACTGACTGACTGACTGACTGACTGACTGACTGACTGACTGACTGACTG**CACTCCATCGAGATTT | 65 |
| B-Catenin110_extF^c^ | **CTGACTG**TGGACTCTGGAATCCATT | 25 |
| B-Catenin133_extR^c^ | **GACTGACTGACTGACTGACTGACTGACTGACTGACTG**TGCCTTTACCACTCAGAG | 55 |
| B-Catenin134_extR^c^ | **CTGACTGACTGACTGACTGACTGACTG**TTGCCTTTACCACTCAGA | 45 |
| GNA11_ext626R | **TGACTGACTGACTGACTGACTGACTGACTGACTGACTGACTGACTG**cttcctccgctccgaccgc | 65 |
| GNAQ626_extF | **TGACTGACTGACTGACTGACTGACTGACTGACTGACTGACTGAC**TGGTCGATGTAGGGGGCC | 62 |
| KIT1669_extF | CAGAAACCCATGTATGAAGTACAG | 24 |
| KIT1676_extF | **ACTGACTGA**ATGTATGAAGTACAGTGGAAGG | 31 |
| KIT1727_extF | **ACTGACTGACTGACTG**TTTACATAGACCCAACACAAC | 37 |
| KIT1924_extF | **GACTGACTGACTGACTGACTGACTG**AAGCCCTCATGTCTGAACTC | 45 |
| KIT2446_extF | **GACTGACTGACTGACTGACTGACTGACTGACTGAC**GATTTTGGTCTAGCCAGA | 53 |
| NRAS34_extR^c^ | **GACTGACT**GCTTTTCCCAACACCAC | 25 |
| NRAS35_extF^c^ | **CTGACTGACTGACTGACTGACTGACTGACTGACTGACTGACTGACTGACTGACTGA**GTGGTGGTTGGAGCAG | 72 |
| NRAS37_extR^c^ | **GACTGACTGACTGACTGACTGACTGACTGACTGACTGACTGACTGACT**CGCTTTTCCCAACAC | 63 |
| NRAS38_extR^c^ | **ACTGACTGACTGACTGACTG**GCGCTTTTCCCAACA | 35 |
| NRAS181_extF^c^ | **GACTGACTGACTGACTGACTGACTGACTGACTGAC**ACATACTGGATACAGCTGGA | 55 |
| NRAS182_extF^c^ | **CTGACTGACTGACTGACTGACTGACTGACTGACTG**CATACTGGATACAGCTGGAC | 55 |
| NRAS183_extR^c^ | **GACTGACTGACTGACTGACTGACTGACTGACTGACTGACTGACTGACTG**CTCATGGCACTGTACTCTTC | 69 |

^a^Primers were purified by polyacrylamide gel electrophoresis.

^b^The sequences are shown 5’>3’ and bold nucleotides are repetitive GACT sequence used to adjust product size.

^c^Primer sequences were published previously.([1](#_ENREF_1))

**Primer Solution Preparation**

***All of the following procedures are performed in PCR hood of Area 1***

**Multiplex PCR Primer Preparation**

1. **Multiplex PCR primer stocks (100 µM).** Multiplex PCR primers are diluted to 100 µM with nuclease-free water upon receipt. After adding water, the primers should remain in the PCR hood for several hours to allow the lyophilized primers to thaw. Mix the primer stocks thoroughly via several rounds of vortexing, and then spin down briefly. Label all primer stocks with names and dates and store at -20 ºC.

***Note:*** *adding 10 fold buffer (µL) based on the nmol number of the primer will make the final concentration 100 µM. For example, if the amount of your primer is 30.5 nmoles, adding 305 µL of water will make the primer final concentration 100 µM.*

1. **Multiplex PCR primer working solutions (3 µM).** For each primer pair, mix the forward and reverse primer (100 µM primer stocks) with nuclease-free water to a final concentration of 3 µM. Mix thoroughly by vortexing and spin down briefly.

Gene A_Exon a_Foward primer stock (100 µM): 30 µL

Gene A_Exon a_Reverse primer stock (100 µM): 30 µL

Nuclease-free H2O: 940 µL

Total volume: 1000 µL

***Note:*** *In order to prevent contamination from multiple openings and closings of the*

*working solution tubes, aliquot the working solutions into 20 small centrifuge tubes, 50 µL/tube, to allow for each single use. Store at -20 ºC.*

1. **PCR primer pools.** Using the following tables, prepare multiplex PCR primer pools for each panel using the 3 µM working solutions. Mix all PCR primers and nuclease-free water by vortexing, and then spin down briefly.

**PCR primer Pool Preparation**

**Table 4. Panel I**

| **Melanoma Panel I - PCR Primers (forward and reverse)** | **[Stock] (**µM**)** | **Volume for 1400 µL (µL)** | **Volume for 350 µL (µL)** | **Volume for 70 µL (µL)** |
| --- | --- | --- | --- | --- |
| NRAS_ex_2 | 3 | 100 | 25 | 5 |
| BRAF_ex_15 | 3 | 100 | 25 | 5 |
| NRAS_ex_3 | 3 | 100 | 25 | 5 |
| Nuclease-Free dH_2_O | - | 1100 | 275 | 55 |

**Table 5. Panel II**

| **Melanoma Panel II - PCR Primers (forward and reverse)** | **[Stock] (**µM**)** | **Volume for 1400 µL (µL)** | **Volume for 350 µL (µL)** | **Volume for 70 µL (µL)** |
| --- | --- | --- | --- | --- |
| KIT557-576 | 3 | 200 | 50 | 10 |
| NRAS_ex_2 | 3 | 100 | 25 | 5 |
| B-Catenin_ex_3 | 3 | 100 | 25 | 5 |
| BRAF_ex_15 | 3 | 100 | 25 | 5 |
| Nuclease-Free dH_2_O | - | 900 | 225 | 45 |

**Table 6. Panel III**

| **Melanoma Panel III - PCR Primers (forward and reverse)** | **[Stock] (**µM**)** | **Volume for 1400 µL (µL)** | **Volume for 350 µL (µL)** | **Volume for 70 µL (µL)** |
| --- | --- | --- | --- | --- |
| NRAS_ex_2 | 3 | 100 | 25 | 5 |
| KIT642 | 3 | 100 | 25 | 5 |
| NRAS_ex_ 3 | 3 | 100 | 25 | 5 |
| GNA11ex5 | 3 | 100 | 25 | 5 |
| Nuclease-Free dH_2_O | - | 1000 | 250 | 50 |

**Table 7. Panel IV**

| **Melanoma Panel IV - PCR Primers (forward and reverse)** | **[Stock] (**µM**)** | **Volume for 1400 µL (µL)** | **Volume for 350 µL (µL)** | **Volume for 70 µL (µL)** |
| --- | --- | --- | --- | --- |
| B-Catenin_ex_3 | 3 | 200 | 50 | 10 |
| BRAF_ex_15 | 3 | 100 | 25 | 5 |
| NRAS_ex_2 | 3 | 100 | 25 | 5 |
| NRAS_ex_3 | 3 | 100 | 25 | 5 |
| Nuclease-Free dH_2_O | - | 900 | 225 | 45 |

**Table 8. Panel V**

| **Melanoma Panel V - PCR Primers (forward and reverse)** | **[Stock] (**µM**)** | **Volume for 1400 µL (µL)** | **Volume for 350 µL (µL)** | **Volume for 70 µL (µL)** |
| --- | --- | --- | --- | --- |
| KIT557-576 | 3 | 400 | 100 | 20 |
| KIT_ex17 | 3 | 400 | 100 | 20 |
| GNAQex5 | 3 | 200 | 50 | 10 |
| Nuclease-Free dH_2_O | - | 400 | 100 | 20 |

***Note:*** *In order to prevent contamination from multiple openings and closings of the primer pool tubes, aliquot the PCR primer pools into small centrifuge tubes, 70 µL/tube (for ~20 sample, to allow for single use. Store at 4 ºC.*

**Extension Primer Preparation**

1. **Extension primer stocks (10 µM or 50 µM).** Add nuclease-free water to dissolve the extension primer to 10 µM or 50 µM. The extension primers should remain in the PCR hood for several hours to allow the lyophilized primers to thaw. Mix the extension primer stocks thoroughly via several rounds of vortexing, and then spin down briefly. Label all primer stocks with names and dates and store at -20 ºC.

***Notes:***

- *Adding 100 fold water (µL) based on the nmol number of the extension primer will make the final concentration 10 µM. For example, if the amount of your primer is 9.5 nmoles, adding 950 µL of water will make the final concentration 10 µM. Adding 20 fold water (µL) based on the nmol number of the extension primer will make the final concentration 50 µM. For example, if the amount of your primer is 9.5 nmoles, adding 190 µL of water will make the final concentration 50 µM.*
- *Most of the original tubes holding the extension primers can only hold a maximum volume of 2000 µL of solution. If you have to add more than 1000 µL of water to make the final concentration to 10 µM, prepare the sample at 50 µM.*

1. **Extension primer working solutions (variable µM as listed below)**. In order to prevent contamination from multiple opening and closing of the extension primer stock solution tubes, aliquot the above extension primers into small tubes, 30-50 µL/tube as delineated below, to allow for single use. Store at -20 ºC.

**Single Base Extension Primer Working Solution- Aliquot Stock- (not pools yet!!!)**

**Table 9. Panel I**

| **MELANOMA PANEL I Extension Primers** | **Desired Working Aliquot Primer Stock (µM)** | **Primer Stock**  **(µM)** | **Primer Stock**  **(µL)** | **MQW (µL)** | **Total in Aliquot (µL)** |
| --- | --- | --- | --- | --- | --- |
| NRAS38_extR | 5 | 50 | 3 | 27 | 30 |
| BRAF1799_extF | 2 | 10 | 6 | 24 | 30 |
| NRAS182_extF | 2 | 10 | 6 | 24 | 30 |
| BRAF1800_extR | 5 | 50 | 5 | 45 | 50 |

**Table 10. Panel II**

| **MELANOMA PANEL II Extension Primers** | **Desired Working Aliquot Primer Stock (µM)** | **Primer Stock**  **(µM)** | **Primer Stock**  **(µL)** | **MQW (µL)** | **Total in Aliquot (µL)** |
| --- | --- | --- | --- | --- | --- |
| KIT1676_extF | 2.5 | 50 | 2 | 38 | 40 |
| B-Catenin133_extR | 5 | 10 | 15 | 15 | 30 |
| NRAS35_extF | 5 | 10 | 15 | 15 | 30 |
| BRAF1799_extR | 2.5 | 50 | 2.5 | 47.5 | 50 |

**Table 11. Panel III**

| **MELANOMA PANEL III Extension Primers** | **Desired Working Aliquot Primer Stock (µM)** | **Primer Stock**  **(µM)** | **Primer Stock**  **(µL)** | **MQW (µL)** | **Total in Aliquot (µL)** |
| --- | --- | --- | --- | --- | --- |
| NRAS34_extR | 10 | 50 | 6 | 24.0 | 30 |
| KIT1924_extF | 2.5 | 50 | 2 | 38.0 | 40 |
| NRAS181_extF | 10 | 10 | 30 | 0.0 | 30 |
| GNA11_ext626R | 2.5 | 10 | 12.5 | 37.5 | 50 |

**Table 12. Panel IV**

| **MELANOMA PANEL IV Extension Primers** | **Desired Working Aliquot Primer Stock (µM)** | **Primer Stock**  **(µM)** | **Primer Stock**  **(µL)** | **MQW (µL)** | **Total in Aliquot (µL)** |
| --- | --- | --- | --- | --- | --- |
| B-Catenin110_extF | 2 | 50 | 2 | 48 | 50 |
| BRAF1798_extF | 2 | 50 | 2 | 48 | 50 |
| B-Catenin134_extR | 2 | 10 | 6 | 24 | 30 |
| NRAS37_extR | 10 | 10 | 30 | 0 | 30 |
| NRAS183_extR | 10 | 10 | 30 | 0 | 30 |

**Table 13. Panel V**

| **MELANOMA PANEL V Extension Primers** | **Desired Working Aliquot Primer Stock (µM)** | **Primer Stock**  **(µM)** | **Primer Stock**  **(µL)** | **MQW (µL)** | **Total in Aliquot (µL)** |
| --- | --- | --- | --- | --- | --- |
| KIT1669_extF | 2.5 | 50 | 2 | 38 | 40 |
| KIT1727_extF | 2.5 | 50 | 2 | 38 | 40 |
| KIT2446_extF | 2.5 | 10 | 10 | 30 | 40 |
| GNAQ626_extF | 5.0 | 10 | 20 | 20 | 40 |

1. **Extension primer pools.** Mix each extension primer using the following tables.

**Single Base Extension Primer Pools**

**Table 14. Panel I**

| **MELANOMA PANEL I Extension Primers** | **Working Aliquot Primer Stock (µM)** | **Volume (µL)**  **(14 µL Total)** | **Volume (µL)**  **(406 µL Total)** | **SBE primer**  **Length (nt)** | **Final [primer] (µM)** |
| --- | --- | --- | --- | --- | --- |
| NRAS38_extR | 5 | 1.0 | 29.0 | 35 | 0.36 |
| BRAF1799_extF | 2 | 2.0 | 58.0 | 45 | 0.29 |
| NRAS182_extF | 2 | 1.5 | 43.5 | 55 | 0.21 |
| BRAF1800_extR | 5 | 8.0 | 232.0 | 65 | 2.9 |
| Nuclease-Free dH2O | - | 1.5 | 43.5 |  |  |

**Table 15. Panel II**

| **MELANOMA PANEL II Extension Primers** | **Working Aliquot Primer Stock (µM)** | **Volume (µL)**  **(16 µL Total)** | **Volume (µL)**  **(400 µL Total)** | **SBE primer**  **Length (nt)** | **Final [primer] (µM)** |
| --- | --- | --- | --- | --- | --- |
| KIT1676_extF | 2.5 | 2.1 | 52.5 | 31 | 0.3 |
| B-Catenin133_extR | 5 | 4.2 | 105.0 | 55 | 1.3 |
| NRAS35_extF | 5 | 1.3 | 32.5 | 72 | 0.4 |
| BRAF1799_extR | 2.5 | 8.4 | 210.0 | 45 | 1.3 |
| Nuclease-Free dH2O | - | 0 |  |  |  |

**Table 16. Panel III**

| **MELANOMA PANEL III Extension Primers** | **Working Aliquot Primer Stock (µM)** | **Volume (µL)**  **(16 µL Total)** | **Volume (µL)**  **(400 µL Total)** | **SBE primer**  **Length (nt)** | **Final [primer] (µM)** |
| --- | --- | --- | --- | --- | --- |
| NRAS34_extR | 10 | 1.0 | 25 | 25 | 0.625 |
| KIT1924_extF | 2.5 | 0.7 | 17.5 | 45 | 0.109 |
| NRAS181_extF | 10 | 2.3 | 57.5 | 55 | 1.438 |
| GNA11_ext626R | 2.5 | 2.1 | 52.5 | 65 | 0.328 |
| Nuclease-Free dH2O | - | 9.9 | 247.5 |  |  |

**Table 17. Panel IV**

| **MELANOMA PANEL IV Extension Primers** | **Working Aliquot Primer Stock (µM)** | **Volume (µL)**  **(16 µL Total)** | **Volume (µL)**  **(400 µL Total)** | **SBE primer**  **Length (nt)** | **Final [primer] (µM)** |
| --- | --- | --- | --- | --- | --- |
| B-Catenin110_extF | 2 | 3.0 | 75 | 25 | 0.375 |
| BRAF1798_extF | 2 | 1.0 | 25 | 35 | 0.125 |
| B-Catenin134_extR | 2 | 1.8 | 45 | 45 | 0.23 |
| NRAS37_extR | 10 | 5.6 | 140 | 63 | 3.5 |
| NRAS183_extR | 10 | 0.8 | 20 | 69 | 0.5 |
| Nuclease-Free dH2O | - | 3.8 | 95 |  |  |

**Table 18. Panel V**

| **MELANOMA PANEL V Extension Primers** | **Working Aliquot Primer Stock (µM)** | **Volume (µL)**  **(16 µL Total)** | **Volume (µL)**  **(400 µL Total)** | **SBE primer**  **Length (nt)** | **Final [primer] (µM)** |
| --- | --- | --- | --- | --- | --- |
| KIT1669_extF | 2.5 | 2.0 | 50 | 24 | 0.31 |
| KIT1727_extF | 2.5 | 1.4 | 35 | 37 | 0.22 |
| KIT2446_extF | 2.5 | 2.0 | 50 | 53 | 0.31 |
| GNAQ626_extF | 5.0 | 4.4 | 110 | 62 | 1.375 |
| Nuclease-Free dH2O | - | 6.2 | 155 | - | - |

***Note:*** *In order to prevent contamination from multiple openings and closings of the extension primer pool tubes, aliquot the extension primer pools into small centrifuge tubes, 25 µL/tube (for ~20 samples), to allow for single use. Store at 4 ºC.*

**SNaPShot genotyping Procedures**

1. **Multiplex PCR**

****The following procedures are performed in the PCR hood of Area 1.***

1. **Clean PCR Hood**. Clean the PCR hood and the bench areas adjacent to the hood with 10% bleach, 70% alcohol and RNase Away.
2. **PCR Master Mix Preparation**. The following lists reagent volumes for one reaction. The total reaction volume for all samples run with five panels will be calculated as, “number of samples (including one positive and one negative control) × 5 panels, plus some extra for pipetting error”. Mix all the reagents in one eppendorf tube, vortex and spin down.

| **Reagents** | **µL per reaction** |
| --- | --- |
| **10× PCR Buffer** | 1 |
| **MgCl_2_ (50 mM)** | 0.6 |
| **dNTPs (2mM)** | 1.5 |
| **Taq** | 0.1 |
| **Total** | 3.2 |

1. **PCR Final Mix Preparation.** Prepare one PCR final mix for each panel. The table below shows the volume of one reaction for one panel. The total number of reactions will be calculated as, the number of samples (including a positive and negative control) plus some extra for pipetting error.

| **Reagents** | **µL per reaction** |
| --- | --- |
| **PCR Master Mix** | 3.2 |
| **PCR Primer Pool X** | 2.8 |
| **Total** | 6.0 |

1. **PCR Reaction Preparation.** Add 6 µL of above PCR final mix into a PCR tube, one PCR strip tube for one sample and one panel per tube. See details in Figure 1.

**Figure 1**

1. **Negative Control.** Add 4 µL of nuclease-free water into the sample strip used as the negative control (see Figure 2). Cover the strip tubes with strip lids.
2. **Cover all the strip tubes with strip lids and move to Area 2.**

****The following procedures are performed in Area 2.***

1. **Add 4 µL of sample (5ng/µL) into its corresponding strip tube.**

**Figure 2**

Cover all the tubes, vortexing thoroughly and spin briefly. Proceed to Area 3.

****The following procedures are performed in the Area Three***

1. **Run PCR:**

95 ºC for 8 min

(95 ºC for 20 sec; 58 ºC for 30 sec; 72 ºC for 1 min) × 40 cycles

72 ºC for 3 min

Hold at 8 ºC

Spin the tubes briefly before starting the next step.

1. **Exo-SAP-IT Treatment**

**** The following procedures are performed in Area 3***

1. **Add 4 µL of *Exo-SAP-IT* into Each Multiplex-PCR Product.** Mix by vortexing**,** and then spin briefly.
2. **Incubate as follows:**

37 ºC for 15 min

80 ºC for 15 min

Hold at 8 ºC

Spin the tubes briefly before staring the next step. Store samples at -20 ºC (in Area 3) if the next step will not start immediately.

1. **Extension Reaction**

****The following procedures are performed in Area 1.***

1. **Extension Mix Preparation. “**Extension Primer Pool” and “SNaPShot RR mix Working Solution” were prepared as previously described. Each panel will have one specific extension mix. The following table shows the reagents needed for one reaction. The total volume of reagents needed for all reactions per one panel will be calculated by multiplying the number of samples plus some extra for pipetting error.

| **Reagent** | **µL per reaction** |
| --- | --- |
| **SNaPShot RR Mix Working Solution** | **6** |
| **Extension Primer Pool X** | **1** |
| **Total** | **7** |

*Note: RR mix should be protected from light.*

1. **Extension Tubes Preparation.** Prepare strip tubes as described in the PCR section. (Figure 3)
2. **Add 7 µL of above Extension Mix into the Prepared Tubes.** (Figure 3)

**Figure 3**

1. **Cover the tubes with lids and proceed to Area 3.**

****The following procedures are performed in the Area 3.***

1. **Add 3 µL of PCR products from PCR tubes into corresponding extension tubes.** (Figure 4)

**Figure 4**

1. **Run SNaPShot Extension Program:**

96 ºC for 30 sec.

(96 ºC for 10 sec.; 50 ºC for 5 sec.; 60 ºC for 30 sec.) × 25 cycles

Hold at 8 ºC

Spin the tubes briefly before starting the next step.

1. **SAP Treatment for Extension Products**
2. **Add 2 µL of SAP into Each Extension Product.** Mix thoroughly by vortexing and spin briefly.
3. **Incubate as follows:**

37 ºC for 1 h

75 ºC for 15 min

Hold at 8 ºC

Spin briefly before staring the next step. Store the samples at -20 ºC (Area 3) if the next step will not start immediately.

1. **Run Extension Products in the ABI 3730 DNA Analyzer**
2. **Formamide and Size Standard Mix Preparation.** Mix Hi-Di Formamide with GeneScane-120LIZ size standard together as indicated below. Calculate the total volume by multiplying the total number of samples.

| **Reagent** | **µL per reaction** |
| --- | --- |
| **Hi-Di Formamide** | **9.3** |
| **GeneScan-120LIZ Size Standard** | **0.2** |

1. **Add 9.5 µL of above Hi-Di/LIZ Mix into the sample wells of a 96-well Plate.** The ABI 3730 analyzer will run odd number columns first, followed by even number columns. So, add samples in the odd number columns first, and then proceed with the even number columns, if necessary.
2. **Add 0.5 µL of SAP Treated Extension Products into the sample wells of the 96-well Plate.**
3. **Add 10 µL of Hi-Di Formamide into each “blank” well of the 96-well Plate.** If you do not have enough samples to fill half of the 96-well plate (odd columns) or the entire 96-well plate (odd and even columns) plate, please fill the blank wells with 10 µL of Hi-Di formamide.
4. **Cover and Vortex the Plate.** Cover the plate with plate sealing film. Vortex the plate and then spin briefly.

*Note: If the following analysis could not be processed immediately, please cover the plate with foil and store at -20 ºC (in Area 3).*

1. **Denature the samples as follows:**

94 ºC for 5 min

Hold at 4 ºC

Put the denatured samples in -20 ºC freezer for 5 min. Then spin the plate briefly.

1. **Cover Plate with a Septa Mat.** Remove the plate sealing film carefully and cover the plate with a septa mat.
2. **Load the plate into ABI 3730 Analyzer.**

**Appendix A: Spiking Primers and Positive Controls**

**Table 19. Spiking primers used for pan-positive control assay**

| **Spiking primer name** | **Primer sequence^a^** | **pmol/ul** |
| --- | --- | --- |
| SspiBRAF1799T>A | aggtgattttggtctagctacagAgaaatctcgatggagtgaaaa | 0.05 |
| AspiBRAF1799T>A | ccactccatcgagatttcTctgtagctagaccaaatcacctaaaa | 0.05 |
| SspiBRAF1799T>G | aggtgattttggtctagctacagGgaaatctcgatggagtgaaaa | 0.05 |
| AspiBRAF1799T>G | ccactccatcgagatttcCctgtagctagaccaaatcacctaaaa | 0.05 |
| SspiBRAF1798_1799GT>AA | aggtgattttggtctagctacaAAgaaatctcgatggagtgaaaa | 0.05 |
| AspiBRAF1798_1799GT>AA | ccactccatcgagatttcTTtgtagctagaccaaatcacctaaaa | 0.05 |
| SspiBRAF1798G>A | aggtgattttggtctagctacaatgaaatctcgatggagtgaaaa | 0.05 |
| AspiBRAF1798G>A | ccactccatcgagatttcattgtagctagaccaaatcacctaaaa | 0.05 |
| SspiBRAF1798_1799GT>AG | aggtgattttggtctagctacaAGgaaatctcgatggagtgaaaa | 0.05 |
| AspiBRAF1798_1799GT>AG | ccactccatcgagatttcCTtgtagctagaccaaatcacctaaaa | 0.05 |
| SspiBRAF1799_1800TG>AA | aggtgattttggtctagctacagAAaaatctcgatggagtgaaaa | 0.05 |
| AspiBRAF1799_1800TG>AA | ccactccatcgagatttTTctgtagctagaccaaatcacctaaaa | 0.05 |
| SspiBRAF1799_1800TG>AT | aggtgattttggtctagctacagATaaatctcgatggagtgaaaa | 0.0125 |
| AspiBRAF1799_1800TG>AT | ccactccatcgagatttATctgtagctagaccaaatcacctaaaa | 0.05 |
| AspiB-cat110C>G | gtagtggcaccaCaatggattccagagtccaggtaagactaaaaa | 0.05 |
| AspiB-cat110C>T | gtagtggcaccaAaatggattccagagtccaggtaagactaaaaa | 0.05 |
| AspiB-cat110 C>A | gtagtggcaccaTaatggattccagagtccaggtaagactaaaaa | 0.05 |
| SspiB-cat133T>C | cagctcctCctctgagtg gtaaaggcaatcctgagaaaaa | 0.05 |
| SspiB-cat134C>T | cagctccttTtctgagtggtaaaggcaatcctgagaaaaa | 0.05 |
| SspiB-cat134C>A | cagctccttAtctgagtggtaaaggcaatcctgagaaaaa | 0.05 |
| SspiGNA11626A>T | atggtggatgtggggggccTgcggtcggagcggaggaagtaaaaa | 0.05 |
| SspiGNA11626A>C | atggtggatgtggggggccCgcggtcggagcggaggaagtaaaaa | 0.05 |
| AspiGNAQ626A>C | ATTTTCTTCTCTCTGACCTTGGGCCCCCTACATCGACCATTAAAAA | 0.05 |
| AspikGNAQ626A>T | ATTTTCTTCTCTCTGACCTTAGGCCCCCTACATCGACCATTAAAAA | 0.05 |
| AspiGNAQ626A>G | ATTTTCTTCTCTCTGACCTTCGGCCCCCTACATCGACCATTAAAAA | 0.05 |
| AspiKIT2446G>C | TCATTCTTGATGTGTCTGGCTAGACCAAAATCACAaaaa | 0.05 |
| AspiKIT1676T>A | CTCAACATCCTTCCACTGTACTTCATACATGGGTTaaaa | 0.05 |
| AspiKIT1676T>C | CTCAACAgCCTTCCACTGTACTTCATACATGGGTTaaaa | 0.05 |
| AspiKIT1669T>A | ACCTTCCtCTGTACTTCATACATGGGTTTCTGTaaaa | 0.05 |
| AspiKIT1669T>C | ACCTTCCgCTGTACTTCATACATGGGTTTCTGTaaaa | 0.05 |
| AspiKIT1727T>C | TAAGGAgGTTGTGTTGGGTCTATGTAAACATAATTaaaa | 0.05 |
| AspiKIT1924A>G | GGACTTCGAGTTCAGACATGAGGGCTTCCCGTTCTaaaa | 0.05 |
| S.ctrl_NRAS34G>A^b^ | ACTGGTGGTGGTTGGAGCAAGTGGTGTTGGGAAAAGCGCAAAAAA | 0.05 |
| S.ctrl_NRAS34G>T^b^ | ACTGGTGGTGGTTGGAGCATGTGGTGTTGGGAAAAGCGCAaaaa | 0.05 |
| S.ctrl_NRAS34G>C^b^ | ACTGGTGGTGGTTGGAGCACGTGGTGTTGGGAAAAGCGCAaaaa | 0.05 |
| AspiNRAS 35 G>T | tcccaacaccaActgctcca accaccaccagtttgaaaa | 0.05 |
| S.ctrl_NRAS35G>C^b^ | ACTGGTGGTGGTTGGAGCAGCTGGTGTTGGGAAAAGCGCAAAAAA | 0.05 |
| A.ctrl_NRAS35G>C^b^ | TGCGCTTTTCCCAACACCAGCTGCTCCAACCACCACCAGTAAAAA | 0.05 |
| AspiNRAS 35 G>A | tcccaacaccaTctgctcca accaccaccagtttgaaaaa | 0.05 |
| S.ctrl_NRAS37G>T^b^ | GGTGGTGGTTGGAGCAGGTTGTGTTGGGAAAAGCGCACTGAAAAA | 0.0125 |
| SspiNRAS37G>C | tggagcaggtCgtgttggga aaagcgcactgacaaaaaaa | 0.0125 |
| S.ctrl_NRAS38G>A^b^ | GGTGGTGGTTGGAGCAGGTGATGTTGGGAAAAGCGCACTGAAAAA | 0.05 |
| S.ctrl_NRAS38G>T^b^ | GGTGGTGGTTGGAGCAGGTGTTGTTGGGAAAAGCGCACTGAAAAA | 0.05 |
| S.ctrl_NRAS38G>C^b^ | GGTGGTGGTTGGAGCAGGTGCTGTTGGGAAAAGCGCACTGaaaa | 0.05 |
| AspiNRAS181C>A | actcttcttTtccagctgtatccagtatgtccaacaaaa | 0.05 |
| AspiNRAS181C>G | actcttcttCtccagctgtatccagtatgtccaacaaaa | 0.05 |
| AspiNRAS182A>T | actcttctAgtccagctgtatccagtatgtccaacaaaa | 0.05 |
| AspiNRAS182A>C | actcttctGgtccagctgtatccagtatgtccaacaaaa | 0.05 |
| AspiNRAS182_183AA>GG | actcttcCCgtccagctgtatccagtatgtccaacaaaa | 0.05 |
| SspiNRAS182_183AA>GG | agctggacGGgaagagtacagtgccatgagagaccaaaaa | 0.05 |
| SspiNRAS183A>C | agctggacaCgaagagtacagtgccatgagagaccaaaaa | 0.05 |
| SspiNRAS183A>T | agctggacaTgaagagtacagtgccatgagagaccaaaaaa | 0.05 |

^a^The sequences are shown 5’>3’ and point mutations are shaded.

^b^Primer sequences were published previously.([1](#_ENREF_1))

***Notes:***

- Four or five ‘A’s were added at the 3’ end of each spiking primer to prevent the spiking primer from working as an extension primer.
- Spiking primers should be added into reactions right after the PCR products are cleaned-up and before the extension step.
- Each spiking primer is used as follows: 1ul of spiking primer (at 0.05pmol/ul- unless otherwise noted above) added to 3 ul of wild type genomic male DNA PCR product.

**Table 20. Pan Positive Control Mix Preparation**

| **Panel** | **Spiking primer** | **Working solution (µM)** | **Volume added (µL)** |
| --- | --- | --- | --- |
| I | S.ctrl_NRAS38G>A | 0.5 | 5 |
|  | S.ctrl_NRAS38G>C | 0.5 | 5 |
|  | S.ctrl_NRAS38G>T | 0.5 | 5 |
|  | AspikeBRAF1799T>A | 0.5 | 5 |
|  | AspikeBRAF1799T>G | 0.5 | 5 |
|  | AspikeNRAS182_183 AA>GG | 0.5 | 5 |
|  | AspikeNRAS182A>T | 0.5 | 5 |
|  | AspikeNRAS182 A>C | 0.5 | 5 |
|  | SspikeBRAF1799_1800TG>AA | 0.5 | 2 |
|  | SspikeBRAF1799_1800TG>AT | 0.5 | 2 |
| II | AspikeKIT1676T>A | 0.5 | 5 |
|  | AspikeKIT1676T>C | 0.5 | 5 |
|  | SspikeBRAF1799T>A | 0.5 | 5 |
|  | SspikeBRAF1799T>G | 0.5 | 5 |
|  | SspikeB-cat133 T>C | 0.5 | 5 |
|  | AspikeNRAS 35 G>A | 0.5 | 5 |
|  | AspikeNRAS 35 G>T | 0.5 | 5 |
|  | A.ctrl_NRAS35G>C | 0.5 | 5 |
| III | S.ctrl_NRAS34G>A | 0.5 | 5 |
|  | S.ctrl_NRAS34G>C | 0.5 | 5 |
|  | S.ctrl_NRAS34G>T | 0.5 | 5 |
|  | AspikeKIT1924A>G | 0.5 | 5 |
|  | AspikeNRAS181C>A | 0.5 | 5 |
|  | AspikeNRAS181C>G | 0.5 | 5 |
|  | GNA11 626A>C Sensespike | 0.5 | 5 |
|  | GNA11 626A>T Sensespike | 0.5 | 5 |
| IV | AspikeB-cat110 C>A | 0.5 | 5 |
|  | AspikeB-cat110 C>G | 0.5 | 5 |
|  | AspikeB-cat110 C>T | 0.5 | 5 |
|  | AspikeBRAF1798G>A | 0.5 | 5 |
|  | SspikeB-cat134 C>A | 0.5 | 5 |
|  | SspikeB-cat134 C>T | 0.5 | 5 |
|  | SspikeNRAS 37 G>C | 0.5 | 2 |
|  | S.ctrl_NRAS37G>T | 0.5 | 2 |
|  | SspikeNRAS183 A>C | 0.5 | 5 |
|  | SspikeNRAS183 A>T | 0.5 | 5 |
|  | SspikeNRAS182_183 AA>GG | 0.5 | 5 |
| V | AspikeKIT1669T>A | 0.5 | 5 |
|  | AspikeKIT1669T>C | 0.5 | 5 |
|  | AspikeKIT1727T>C | 0.5 | 5 |
|  | AspikeKIT2446G>C | 0.5 | 10 |
|  | AspikeGNAQ626A>C | 0.5 | 5 |
|  | AspikeGNAQ626A>T | 0.5 | 5 |
|  | AspikeGNAQ626A>G | 0.5 | 5 |

***Note:*** 1 ul of Pan Positive Mix is added into 2.5 ul of human genomic DNA PCR product for extension reaction.

**Appendix B: SNaPshot Screen Figures**


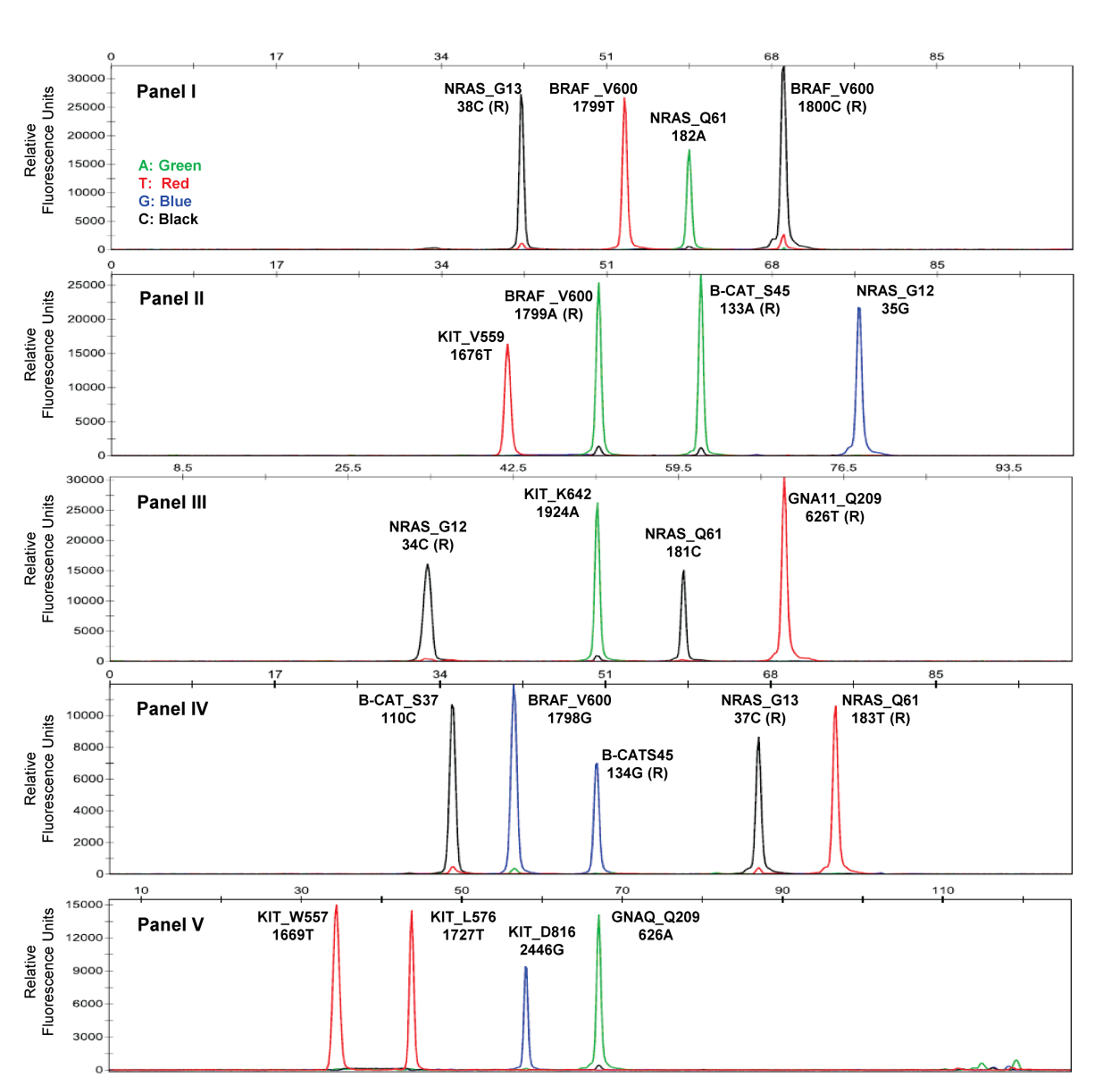


**Figure 5. Melanoma SNaPshot screen using wildtype DNA.** Five multiplexed panels can detect the mutational status of twenty gene loci. Each peak color represents a particular nucleotide at that locus. The gene name, amino acid, and nucleotide are labeled above each peak. An “(R)” after the nucleotide denotes a reverse extension primer.


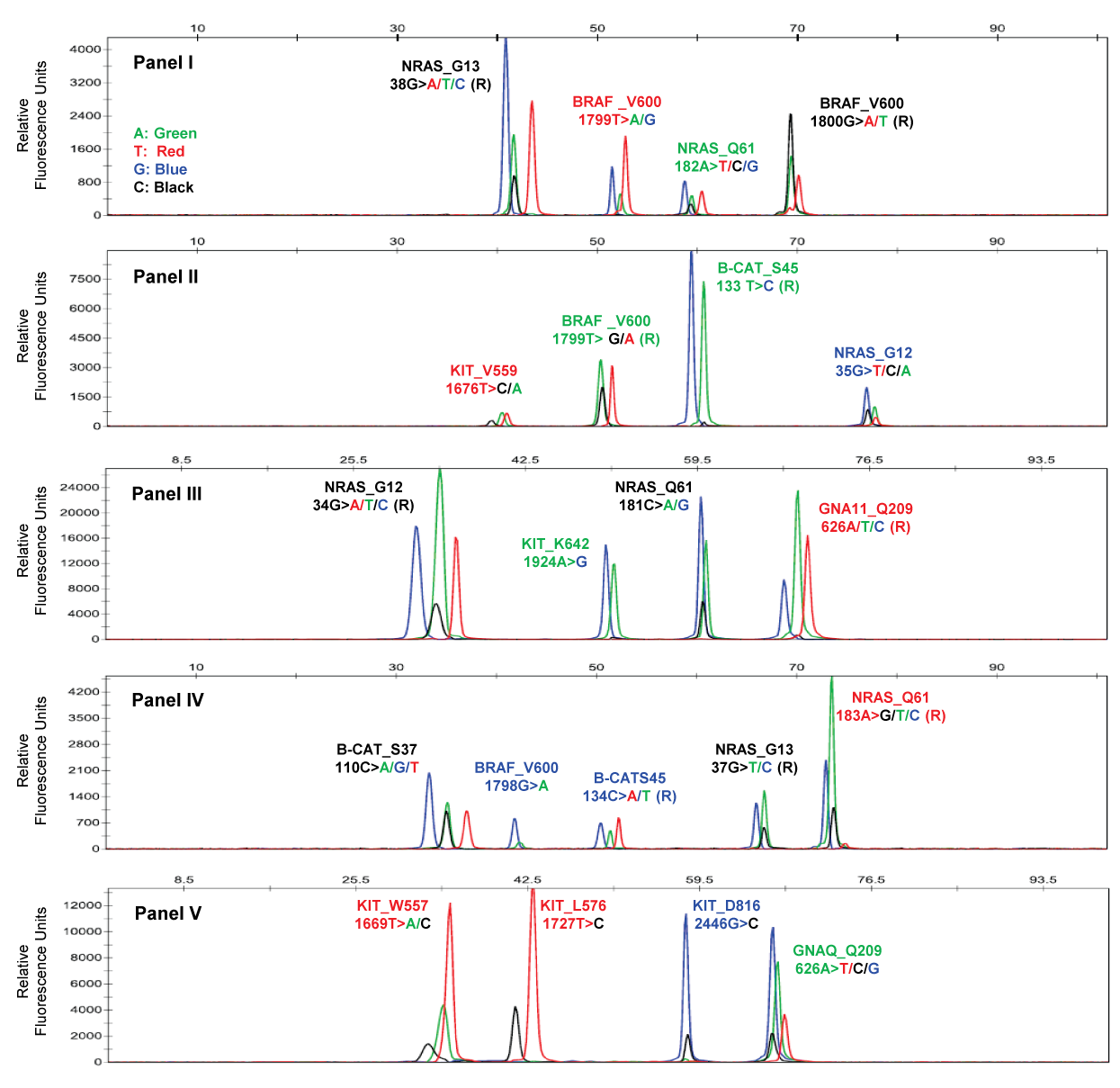


**Figure 6. Melanoma SNaPshot screen using pan positive controls.** Five multiplexed panels can detect the mutational status of twenty gene loci. Each peak color represents a particular nucleotide at that locus. The gene name, amino acid, and nucleotide are labeled above each peak. An “(R)” after the nucleotide denotes a reverse extension primer.

**REFERENCES**

1. Dias-Santagata D, Akhavanfard S, David SS, Vernovsky K, Kuhlmann G, Boisvert SL, et al. Rapid targeted mutational analysis of human tumours: a clinical platform to guide personalized cancer medicine. EMBO Mol Med. 2010;2:146-58.

2. Van Raamsdonk CD, Bezrookove V, Green G, Bauer J, Gaugler L, O'Brien JM, et al. Frequent somatic mutations of GNAQ in uveal melanoma and blue naevi. Nature. 2009;457:599-602.
