# Supplementary material for: Routine Multiplex Mutational Profiling of Melanomas Enables Enrollment in Genotype-Driven Therapeutic Trials
Source: PLoS One. 2012 Apr 20;7(4):e35309. doi: 10.1371/journal.pone.0035309 (PMC3335021; doi:10.1371/journal.pone.0035309)
Supplement: Table S1 — PCR primers for SNaPshot screen. (DOC) [file pone.0035309.s005.doc]

**Table S1.** PCR primers for SNaPshot screen.

| **Amplification primer**  **name** | **Primer sequence^a^** | **Length of product (bp)** |
| --- | --- | --- |
| BRAF_ex15_a1^b^ | ACGTTGGATGTGCTTGCTCTGATAGGAAAATG | 143 |
| BRAF_ex15_a2^b^ | ACGTTGGATGCTGATGGGACCCACTCCAT | 143 |
| β-Catenin_ex3_a1^b^ | ACGTTGGATGTCACTGGCAGCAACAGTCTT | 89 |
| β-Catenin_ex3_a2^b^ | ACGTTGGATGCAGGATTGCCTTTACCACTCA | 89 |
| GNA11ex5F | TGCAGATTGGGCCTTGGGGC | 197 |
| GNA11ex5R | GCAGGGCCCACCTCGTTGTC | 197 |
| GNAQex5A^c^ | CCCACACCCTACTTTCTATCATTTAC | 298 |
| GNAQex5B^c^ | TTTTCCCTAAGTTTGTAAGTAGTGC | 298 |
| KIT642F | GCGGCCATGACTGTCGCTGT | 251 |
| KIT642R | AGGCAGCTTGGACACGGCTT | 251 |
| KIT557-576F | TCTCCAGAGTGCTCTAATGACTGAGAC | 189 |
| KIT557-576R | GCCTGTTTCTGGGAAACTCCCATT | 189 |
| KIT_ex17_a1^b^ | ACGTTGGATGTCATGGTCGGATCACAAAGA | 98 |
| KIT_ex17_a2^b^ | ACGTTGGATGGAGAATGGGTACTCACGTTTCC | 98 |
| NRAS_ex2_a1^b^ | ACGTTGGATGCAACAGGTTCTTGCTGGTGT | 175 |
| NRAS_ex2_a2^b^ | ACGTTGGATGGAGAGACAGGATCAGGTCAGC | 175 |
| NRAS_ex3_a1^b^ | ACGTTGGATGTGGTGAAACCTGTTTGTTGG | 179 |
| NRAS_ex3_a2^b^ | ACGTTGGATGCCTTTCAGAGAAAATAATGCTCCT | 179 |

^a^The sequences are shown 5’>3’.

^b^Primer sequences were published previously.

^c^Primer sequences were published previously.
