# Supplementary material for: Routine Multiplex Mutational Profiling of Melanomas Enables Enrollment in Genotype-Driven Therapeutic Trials
Source: PLoS One. 2012 Apr 20;7(4):e35309. doi: 10.1371/journal.pone.0035309 (PMC3335021; doi:10.1371/journal.pone.0035309)
Supplement: Table S2 — Single-base extension primers for SNaPshot screen. (DOC) [file pone.0035309.s006.doc]

**Table S2.** Single-base extension primers for SNaPshot screen.

| **Extension primer**  **name^a^** | **Primer sequence^b^** | **Primer length (nucleotides)** |
| --- | --- | --- |
| BRAF1798_extF | **CTGACTGACTGACT**GGTGATTTTGGTCTAGCTACA | 35 |
| BRAF1799_extF^c^ | **GACTGACTGACTGACTGACTGACT**GTGATTTTGGTCTAGCTACAG | 45 |
| BRAF1799_extR | **ACTGACTGACTGACTGACTGACTGACTG**CACTCCATCGAGATTTC | 45 |
| BRAF1800_extR | **GACTGACTGACTGACTGACTGACTGACTGACTGACTGACTGACTGACTG**CACTCCATCGAGATTT | 65 |
| β-Catenin110_extF^c^ | **CTGACTG**TGGACTCTGGAATCCATT | 25 |
| β-Catenin133_extR^c^ | **GACTGACTGACTGACTGACTGACTGACTGACTGACTG**TGCCTTTACCACTCAGAG | 55 |
| β-Catenin134_extR^c^ | **CTGACTGACTGACTGACTGACTGACTG**TTGCCTTTACCACTCAGA | 45 |
| GNA11_ext626R | **TGACTGACTGACTGACTGACTGACTGACTGACTGACTGACTGACTG**cttcctccgctccgaccgc | 65 |
| GNAQ626_extF | **TGACTGACTGACTGACTGACTGACTGACTGACTGACTGACTGAC**TGGTCGATGTAGGGGGCC | 62 |
| KIT1669_extF | CAGAAACCCATGTATGAAGTACAG | 24 |
| KIT1676_extF | **ACTGACTGA**ATGTATGAAGTACAGTGGAAGG | 31 |
| KIT1727_extF | **ACTGACTGACTGACTG**TTTACATAGACCCAACACAAC | 37 |
| KIT1924_extF | **GACTGACTGACTGACTGACTGACTG**AAGCCCTCATGTCTGAACTC | 45 |
| KIT2446_extF | **GACTGACTGACTGACTGACTGACTGACTGACTGAC**GATTTTGGTCTAGCCAGA | 53 |
| NRAS34_extR^c^ | **GACTGACT**GCTTTTCCCAACACCAC | 25 |
| NRAS35_extF^c^ | **CTGACTGACTGACTGACTGACTGACTGACTGACTGACTGACTGACTGACTGACTGA**GTGGTGGTTGGAGCAG | 72 |
| NRAS37_extR^c^ | **GACTGACTGACTGACTGACTGACTGACTGACTGACTGACTGACTGACT**CGCTTTTCCCAACAC | 63 |
| NRAS38_extR^c^ | **ACTGACTGACTGACTGACTG**GCGCTTTTCCCAACA | 35 |
| NRAS181_extF^c^ | **GACTGACTGACTGACTGACTGACTGACTGACTGAC**ACATACTGGATACAGCTGGA | 55 |
| NRAS182_extF^c^ | **CTGACTGACTGACTGACTGACTGACTGACTGACTG**CATACTGGATACAGCTGGAC | 55 |
| NRAS183_extR^c^ | **GACTGACTGACTGACTGACTGACTGACTGACTGACTGACTGACTGACTG**CTCATGGCACTGTACTCTTC | 69 |

^a^Primers were purified by polyacrylamide gel electrophoresis.

^b^The sequences are shown 5’>3’ and bold nucleotides are repetitive GACT sequence used to adjust product size.

^c^Primer sequences were published previously.
