# Supplementary material for: Routine Multiplex Mutational Profiling of Melanomas Enables Enrollment in Genotype-Driven Therapeutic Trials
Source: PLoS One. 2012 Apr 20;7(4):e35309. doi: 10.1371/journal.pone.0035309 (PMC3335021; doi:10.1371/journal.pone.0035309)
Supplement: Table S3 — Spiking primers used for pan-positive control assay. (DOC) [file pone.0035309.s007.doc]

**Table S3.** Spiking primers used for pan-positive control assay.

| **Spiking primer name** | **Primer sequence^a^** |
| --- | --- |
| SspiBRAF1799T>A | aggtgattttggtctagctacag**A**gaaatctcgatggagtgaaaa |
| AspiBRAF1799T>A | ccactccatcgagatttc**T**ctgtagctagaccaaatcacctaaaa |
| SspiBRAF1799T>G | aggtgattttggtctagctacag**G**gaaatctcgatggagtgaaaa |
| AspiBRAF1799T>G | ccactccatcgagatttc**C**ctgtagctagaccaaatcacctaaaa |
| SspiBRAF1798_1799GT>AA | aggtgattttggtctagctaca**AA**gaaatctcgatggagtgaaaa |
| AspiBRAF1798_1799GT>AA | ccactccatcgagatttc**TT**tgtagctagaccaaatcacctaaaa |
| SspiBRAF1798G>A | aggtgattttggtctagctaca**a**tgaaatctcgatggagtgaaaa |
| AspiBRAF1798G>A | ccactccatcgagatttca**t**tgtagctagaccaaatcacctaaaa |
| SspiBRAF1798_1799GT>AG | aggtgattttggtctagctaca**AG**gaaatctcgatggagtgaaaa |
| AspiBRAF1798_1799GT>AG | ccactccatcgagatttc**CT**tgtagctagaccaaatcacctaaaa |
| SspiBRAF1799_1800TG>AA | aggtgattttggtctagctacag**AA**aaatctcgatggagtgaaaa |
| AspiBRAF1799_1800TG>AA | ccactccatcgagattt**TT**ctgtagctagaccaaatcacctaaaa |
| SspiBRAF1799_1800TG>AT | aggtgattttggtctagctacag**AT**aaatctcgatggagtgaaaa |
| AspiBRAF1799_1800TG>AT | ccactccatcgagattt**AT**ctgtagctagaccaaatcacctaaaa |
| AspiB-cat110C>G | gtagtggcacca**C**aatggattccagagtccaggtaagactaaaaa |
| AspiB-cat110C>T | gtagtggcacca**A**aatggattccagagtccaggtaagactaaaaa |
| AspiB-cat110 C>A | gtagtggcacca**T**aatggattccagagtccaggtaagactaaaaa |
| SspiB-cat133T>C | cagctcct**C**ctctgagtg gtaaaggcaatcctgagaaaaa |
| SspiB-cat134C>T | cagctcctt**T**tctgagtggtaaaggcaatcctgagaaaaa |
| SspiB-cat134C>A | cagctcctt**A**tctgagtggtaaaggcaatcctgagaaaaa |
| SspiGNA11626A>T | atggtggatgtggggggcc**T**gcggtcggagcggaggaagtaaaaa |
| SspiGNA11626A>C | atggtggatgtggggggcc**C**gcggtcggagcggaggaagtaaaaa |
| AspiGNAQ626A>C | ATTTTCTTCTCTCTGACCTT**G**GGCCCCCTACATCGACCATTAAAAA |
| AspikGNAQ626A>T | ATTTTCTTCTCTCTGACCTT**A**GGCCCCCTACATCGACCATTAAAAA |
| AspiGNAQ626A>G | ATTTTCTTCTCTCTGACCTT**C**GGCCCCCTACATCGACCATTAAAAA |
| AspiKIT2446G>C | TCATTCTTGATGT**G**TCTGGCTAGACCAAAATCACAaaaa |
| AspiKIT1676T>A | CTCAACA**T**CCTTCCACTGTACTTCATACATGGGTTaaaa |
| AspiKIT1676T>C | CTCAACA**g**CCTTCCACTGTACTTCATACATGGGTTaaaa |
| AspiKIT1669T>A | ACCTTCC**t**CTGTACTTCATACATGGGTTTCTGTaaaa |
| AspiKIT1669T>C | ACCTTCC**g**CTGTACTTCATACATGGGTTTCTGTaaaa |
| AspiKIT1727T>C | TAAGGA**g**GTTGTGTTGGGTCTATGTAAACATAATTaaaa |
| AspiKIT1924A>G | GGACTT**C**GAGTTCAGACATGAGGGCTTCCCGTTCTaaaa |
| S.ctrl_NRAS34G>A^b^ | ACTGGTGGTGGTTGGAGCA**A**GTGGTGTTGGGAAAAGCGCAAAAAA |
| S.ctrl_NRAS34G>T^b^ | ACTGGTGGTGGTTGGAGCA**T**GTGGTGTTGGGAAAAGCGCAaaaa |
| S.ctrl_NRAS34G>C^b^ | ACTGGTGGTGGTTGGAGCA**C**GTGGTGTTGGGAAAAGCGCAaaaa |
| AspiNRAS 35 G>T | tcccaacacca**A**ctgctcca accaccaccagtttgaaaa |
| S.ctrl_NRAS35G>C^b^ | ACTGGTGGTGGTTGGAGCAG**C**TGGTGTTGGGAAAAGCGCAAAAAA |
| A.ctrl_NRAS35G>C^b^ | TGCGCTTTTCCCAACACCA**G**CTGCTCCAACCACCACCAGTAAAAA |
| AspiNRAS 35 G>A | tcccaacacca**T**ctgctcca accaccaccagtttgaaaaa |
| S.ctrl_NRAS37G>T^b^ | GGTGGTGGTTGGAGCAGGT**T**GTGTTGGGAAAAGCGCACTGAAAAA |
| SspiNRAS 37G>C | tggagcaggt**C**gtgttggga aaagcgcactgacaaaaaaa |
| S.ctrl_NRAS38G>A^b^ | GGTGGTGGTTGGAGCAGGTG**A**TGTTGGGAAAAGCGCACTGAAAAA |
| S.ctrl_NRAS38G>T^b^ | GGTGGTGGTTGGAGCAGGTG**T**TGTTGGGAAAAGCGCACTGAAAAA |
| S.ctrl_NRAS38G>C^b^ | GGTGGTGGTTGGAGCAGGTG**C**TGTTGGGAAAAGCGCACTGaaaa |
| AspiNRAS181C>A | actcttctt**T**tccagctgtatccagtatgtccaacaaaa |
| AspiNRAS181C>G | actcttctt**C**tccagctgtatccagtatgtccaacaaaa |
| AspiNRAS182A>T | actcttct**A**gtccagctgtatccagtatgtccaacaaaa |
| AspiNRAS182A>C | actcttct**G**gtccagctgtatccagtatgtccaacaaaa |
| AspiNRAS182_183AA>GG | actcttc**CC**gtccagctgtatccagtatgtccaacaaaa |
| SspiNRAS182_183AA>GG | agctggac**GG**gaagagtacagtgccatgagagaccaaaaa |
| SspiNRAS183A>C | agctggaca**C**gaagagtacagtgccatgagagaccaaaaa |
| SspiNRAS183A>T | agctggaca**T**gaagagtacagtgccatgagagaccaaaaaa |

^a^The sequences are shown 5’>3’ and point mutations are shaded.

^b^Primer sequences were published previously.
