# Supplementary material for: Routine Multiplex Mutational Profiling of Melanomas Enables Enrollment in Genotype-Driven Therapeutic Trials
Source: PLoS One. 2012 Apr 20;7(4):e35309. doi: 10.1371/journal.pone.0035309 (PMC3335021; doi:10.1371/journal.pone.0035309)
Supplement: Table S4 — Pan-positive control mix preparation. (DOC) [file pone.0035309.s008.doc]

**Table S4.** Pan-positive control mix preparation.

| **Panel** | **Spiking primer** | **Working solution (µM)** | **Volume added (µL)** |
| --- | --- | --- | --- |
| I | S.ctrl_NRAS38G>A | 0.5 | 5 |
| I | S.ctrl_NRAS38G>C | 0.5 | 5 |
| I | S.ctrl_NRAS38G>T | 0.5 | 5 |
| I | AspikeBRAF1799T>A | 0.5 | 5 |
| I | AspikeBRAF1799T>G | 0.5 | 5 |
| I | AspikeNRAS182_183 AA>GG | 0.5 | 5 |
| I | AspikeNRAS182A>T | 0.5 | 5 |
| I | AspikeNRAS182 A>C | 0.5 | 5 |
| I | SspikeBRAF1799_1800TG>AA | 0.5 | 2 |
| I | SspikeBRAF1799_1800TG>AT | 0.5 | 2 |
| II | AspikeKIT1676T>A | 0.5 | 5 |
| II | AspikeKIT1676T>C | 0.5 | 5 |
| II | SspikeBRAF1799T>A | 0.5 | 5 |
| II | SspikeBRAF1799T>G | 0.5 | 5 |
| II | SspikeB-cat133 T>C | 0.5 | 5 |
| II | AspikeNRAS 35 G>A | 0.5 | 5 |
| II | AspikeNRAS 35 G>T | 0.5 | 5 |
| II | A.ctrl_NRAS35G>C | 0.5 | 5 |
| III | S.ctrl_NRAS34G>A | 0.5 | 5 |
| III | S.ctrl_NRAS34G>C | 0.5 | 5 |
| III | S.ctrl_NRAS34G>T | 0.5 | 5 |
| III | AspikeKIT1924A>G | 0.5 | 5 |
| III | AspikeNRAS181C>A | 0.5 | 5 |
| III | AspikeNRAS181C>G | 0.5 | 5 |
| III | GNA11 626A>C Sensespike | 0.5 | 5 |
| III | GNA11 626A>T Sensespike | 0.5 | 5 |
| IV | AspikeB-cat110 C>A | 0.5 | 5 |
| IV | AspikeB-cat110 C>G | 0.5 | 5 |
| IV | AspikeB-cat110 C>T | 0.5 | 5 |
| IV | AspikeBRAF1798G>A | 0.5 | 5 |
| IV | SspikeB-cat134 C>A | 0.5 | 5 |
| IV | SspikeB-cat134 C>T | 0.5 | 5 |
| IV | SspikeNRAS 37 G>C | 0.5 | 2 |
| IV | S.ctrl_NRAS37G>T | 0.5 | 2 |
| IV | SspikeNRAS183 A>C | 0.5 | 5 |
| IV | SspikeNRAS183 A>T | 0.5 | 5 |
| IV | SspikeNRAS182_183 AA>GG | 0.5 | 5 |
| V | AspikeKIT1669T>A | 0.5 | 5 |
| V | AspikeKIT1669T>C | 0.5 | 5 |
| V | AspikeKIT1727T>C | 0.5 | 5 |
| V | AspikeKIT2446G>C | 0.5 | 10 |
| V | AspikeGNAQ626A>C | 0.5 | 5 |
| V | AspikeGNAQ626A>T | 0.5 | 5 |
| V | AspikeGNAQ626A>G | 0.5 | 5 |
