# Supplementary material for: Routine Multiplex Mutational Profiling of Melanomas Enables Enrollment in Genotype-Driven Therapeutic Trials
Source: PLoS One. 2012 Apr 20;7(4):e35309. doi: 10.1371/journal.pone.0035309 (PMC3335021; doi:10.1371/journal.pone.0035309)
Supplement: Table S5 — PCR primers used for direct sequencing. (DOC) [file pone.0035309.s009.doc]

**Table S5.** PCR primers used for direct sequencing.

| **Sequencing primer name** | **Primer sequence^a^** |
| --- | --- |
| Seq_BRAF_ex15_F | **GTTTTCCCAGTCACGAC**TCATAATGCTTGCTCTGATAG A |
| Seq_BRAF_ex15_R | **CAGGAAACAGCTATGAC**GGCCAAAAATTTAATCAGTGGA |
| Seq_B-Catenin_ex3_F | **GTTTTCCCAGTCACGAC**TGGAACCAGACAGAAAAGCGGC |
| Seq_B-Catenin_ex3_R | **CAGGAAACAGCTATGAC**AGCTACTTGTTCTTGAGTGAAGGACTGA |
| Seq_NRAS_ex2_F | **GTTTTCCCAGTCACGAC**ACCAAATGGAAGGTCACACTAGGGTTT |
| Seq_NRAS_ex2_R | **CAGGAAACAGCTATGAC**ACAGGATCAGGTCAGCGGGC |
| Seq_NRAS_ex3_F | **GTTTTCCCAGTCACGAC**TGAGGGACAAACCAGATAGGCAGA |
| Seq_NRAS_ex3_R | **CAGGAAACAGCTATGAC**CCTAGTGTGGTAACCTCATTTCCCCA |
| Seq_KIT_ex11_F | **GTTTTCCCAGTCACGAC**CCAGAGTGCTCTAATGACTG |
| Seq_KIT_ex11_R | **CAGGAAACAGCTATGAC**ACCCAAA AAGGTGACATGGA |
| Seq_KIT_ex13_F | **GTTTTCCCAGTCACGAC**ATCAGTTTGCCAGTTGTGCT |
| Seq_KIT_ex13_R | **CAGGAAACAGCTATGAC**TTTATAATCTAGCATTGCC |
| Seq_GNAQ_ex11_F | **GTTTTCCCAGTCACGAC**CCCACACCCTACTTTCTATCATTTAC |
| Seq_GNAQ_ex11_R | **CAGGAAACAGCTATGAC**TTTTCCCTAAGTTTGTAAGTAGTGC |
| Seq_GNA11_ex5_F | **GTTTTCCCAGTCACGAC**CCTTGCCCGTTCTAAGAGTG |
| Seq_GNA1_ex5_R | **CAGGAAACAGCTATGAC**GAACCAGGGGTAGGTGATGA |

^a^Sequence shown 5’>3’ and the M13 tag is indicated in bold.
