# Supplementary material for: Routine Multiplex Mutational Profiling of Melanomas Enables Enrollment in Genotype-Driven Therapeutic Trials
Source: PLoS One. 2012 Apr 20;7(4):e35309. doi: 10.1371/journal.pone.0035309 (PMC3335021; doi:10.1371/journal.pone.0035309)
Supplement: Table S6 — Summary of mutations detected in cell lines, frozen tissues, and FFPE samples. (DOC) [file pone.0035309.s010.doc]

**Table S6.** Summary of mutations detected in cell lines, frozen tissues, and FFPE samples.

| **Sample Types** | **Genes** | **Amino Acids** | **Nucleotides** | **# of Mutations** |
| --- | --- | --- | --- | --- |
| **Cell Lines** | *BRAF* | p.V600K | c.1798_1799GT>AA | 1 |
| Total #: 16 | *BRAF* | p.V600E | c.1799T>A | 1 |
| Mutations: 10/10 cell lines | *BRAF* | p.V600D | c.1799_1800TG>AT | 1 |
|  | *NRAS* | p.Q61R | c.182A>G | 1 |
|  | *NRAS* | p.Q61K | c.181C>A | 1 |
|  | *NRAS* | p.Q61L | c.182A>T | 1 |
|  | *KIT* | p.L576P | c.1727T>C | 1 |
|  | *GNAQ* | p.Q209P | c.626A>C | 1 |
|  | *GNAQ* | p.Q209L | c.626A>T | 1 |
|  | *CTNNB1* | p.S45F | c.134C>T | 1 |
| **Frozen Tissues** | *BRAF* | p.V600E | c.1799T>A | 10 |
| Total #: 24 | *BRAF* | p.V600K | c.1798_1799GT>AA | 2 |
| Mutations: 17/24 samples | *BRAF* | p.V600M | c.1798G>A | 1 |
|  | *NRAS* | p.G13A | c.37G>C | 1 |
|  | *NRAS* | p.Q61R | c.182A>G | 2 |
|  | *KIT* | p.L576P | c.1727T>C | 1 |
| **FFPE Samples** | *BRAF* | p.V600K | c.1798_1799GT>AA | 2 |
| Total #: 18 | *BRAF* | p.V600E | c.1799T>A | 4 |
| Mutations: 14/17 samples | *BRAF* | p.V600R | c.1798_1799GT>AG | 1 |
|  | *NRAS* | p.G13D | c.38G>A | 1 |
|  | *KIT* | p.W557R | c.1669T>C | 1 |
|  | *KIT* |  | c.1669T>A | 1 |
|  | *KIT* | p.V559A | c.1676T>C | 1 |
|  | *KIT* | p.V559D | c.1676T>A | 1 |
|  | *KIT* | p.L576P | c.727T>C | 1 |
|  | *KIT* | p.K642E | c.1924A>G | 1 |
