# Supplementary material for: Routine Multiplex Mutational Profiling of Melanomas Enables Enrollment in Genotype-Driven Therapeutic Trials
Source: PLoS One. 2012 Apr 20;7(4):e35309. doi: 10.1371/journal.pone.0035309 (PMC3335021; doi:10.1371/journal.pone.0035309)
Supplement: Table S7 — SNaPshot assay results for cell lines. (DOC) [file pone.0035309.s011.doc]

**Table S7.** SNaPshot assay results for cell lines.

| **Cell line** | ***BRAF*** | ***NRAS*** | ***KIT*** | ***CTNNB1*** | ***GNAQ*** | ***GNA11*** | **Tissue derivation** | **Ref^e^** |
| --- | --- | --- | --- | --- | --- | --- | --- | --- |
| WM1361A | WT^d^ | p.Q61R; c.182A>G | WT | WT | WT | WT | Melanoma |  |
| SK-Mel-238 | p.V600K; c.1798_1799 GT>AA | WT | WT | WT | WT | WT | Melanoma |  |
| SK-Mel-90 | WT | p.Q61K; c.181C>A | WT | WT | WT | WT | Melanoma |  |
| H358^a^ | WT | WT | WT | WT | WT | WT | Lung adeno^f^ |  |
| H2009^a^ | WT | WT | WT | WT | WT | WT | Lung adeno |  |
| LoVo^a^ | WT | WT | WT | WT | WT | WT | Colon |  |
| H460^a^ | WT | WT | WT | WT | WT | WT | Lung LCC^g^ |  |
| H1975 | WT | WT | WT | WT | WT | WT | Lung adeno |  |
| H1666^b^ | WT | WT | WT | WT | WT | WT | Lung adeno |  |
| Mel270^c^ | WT | WT | WT | WT | p.Q209P; c.626A>C | WT | Uveal melanoma |  |
| 92.1^c^ | WT | WT | WT | WT | p.Q209L; c.626A>T | WT | Uveal melanoma |  |
| WM115 | p.V600E; c.1799T>A | WT | WT | WT | WT | WT | Melanoma |  |
| WM1963 | WT | WT | WT | p.S45F; c.134C>T | WT | WT | Melanoma |  |
| WM266-4 | p.V600D; c.1799_1800 TG>AT | WT | WT | WT | WT | WT | Melanoma |  |
| WM3211 | WT | WT | p.L576P; c.1727T>C | WT | WT | WT | Melanoma |  |
| WM3682 | WT | p.Q61L; c.182A>T | WT | WT | WT | WT | Melanoma |  |

^a^These cell lines have *KRAS* mutations (H2009 KRAS_G12A; H358 KRAS_G12C; LoVo KRAS_G13D; H460 KRAS_Q61H).

^b^This cell line has a BRAF_G466V mutation.

^c^Whole genome amplified from cell line DNA.

^d^WT; wild type

^e^Ref; reference for previously-identified mutation indicated

^f^Adeno, adenocarcinoma

^g^LCC; large cell carcinoma
