# Supplementary material for: Routine Multiplex Mutational Profiling of Melanomas Enables Enrollment in Genotype-Driven Therapeutic Trials
Source: PLoS One. 2012 Apr 20;7(4):e35309. doi: 10.1371/journal.pone.0035309 (PMC3335021; doi:10.1371/journal.pone.0035309)
Supplement: Table S8 — SNaPshot assay results for fresh-frozen primary human melanomas. (DOC) [file pone.0035309.s012.doc]

**Table S8**. SNaPshot assay results for fresh-frozen primary human melanomas.

| **Sample** | ***BRAF*** | ***NRAS*** | ***KIT*** | ***CTNNB1*** | ***GNAQ*** | ***GNA11*** |
| --- | --- | --- | --- | --- | --- | --- |
| 1 | p.V600E; c.1799T>A | WT^a^ | WT | WT | WT | WT |
| 2 | p.V600E; c.1799T>A | WT | WT | WT | WT | WT |
| 3 | p.V600E; c.1799T>A | WT | WT | WT | WT | WT |
| 4 | p.V600K; c.1798_1799GT>AA | WT | WT | WT | WT | WT |
| 5 | p.V600K; c.1798_1799GT>AA | WT | WT | WT | WT | WT |
| 6 | p.V600M; c.1798G>A | WT | WT | WT | WT | WT |
| 7 | WT | p.Q61R; c.182A>G | WT | WT | WT | WT |
| 8 | p.V600E; c.1799T>A | WT | WT | WT | WT | WT |
| 9 | WT | WT | WT | WT | WT | WT |
| 10 | WT | WT | WT | WT | WT | WT |
| 11 | WT | p.G13A; c.37G>C | WT | WT | WT | WT |
| 12 | WT | WT | WT | WT | WT | WT |
| 13 | p.V600E; c.1799T>A | WT | WT | WT | WT | WT |
| 14 | WT | WT | WT | WT | WT | WT |
| 15 | WT | WT | WT | WT | WT | WT |
| 16 | p.V600E; c.1799T>A | WT | WT | WT | WT | WT |
| 17 | p.V600E; c.1799T>A | WT | WT | WT | WT | WT |
| 18 | WT | WT | p.L576P; c.1727T>C | WT | WT | WT |
| 19 | p.V600E; c.1799T>A | WT | WT | WT | WT | WT |
| 20 | p.V600E; c.1799T>A | WT | WT | WT | WT | WT |
| 21 | WT | WT | WT | WT | WT | WT |
| 22 | WT | WT | WT | WT | WT | WT |
| 23 | WT | p.Q61R; c.182A>G | WT | WT | WT | WT |
| 24 | p.V600E; c.1799T>A | WT | WT | WT | WT | WT |

^a^WT; wildtype
