# Supplementary material for: Routine Multiplex Mutational Profiling of Melanomas Enables Enrollment in Genotype-Driven Therapeutic Trials
Source: PLoS One. 2012 Apr 20;7(4):e35309. doi: 10.1371/journal.pone.0035309 (PMC3335021; doi:10.1371/journal.pone.0035309)
Supplement: Table S9 — SNaPshot assay results for FFPE tissue. (DOC) [file pone.0035309.s013.doc]

**Table S9**. SNaPshot assay results for FFPE tissue.

| **Sample** | **Name** | ***BRAF*** | ***NRAS*** | ***KIT*** | ***CTNNB1*** | ***GNAQ*** | ***GNA11*** |
| --- | --- | --- | --- | --- | --- | --- | --- |
| 1 | OHSU10-1 | WT^a^ | WT | p.W557R; c.1669T>C | WT | WT | WT |
| 2 | OHSU10-2 | WT | WT | p.W557R; c.1669T>A | WT | WT | WT |
| 3 | OHSU10-3 | WT | WT | p.V559A; c.1676T>C | WT | WT | WT |
| 4 | OHSU10-4 | WT | WT | p.V559D; c.1676T>A | WT | WT | WT |
| 5 | OHSU10-5 | WT | WT | p.L576P; c.1727T>C | WT | WT | WT |
| 6 | OHSU10-6 | WT | WT | p.K642E; c.1924A>G | WT | WT | WT |
| 7 | OHSU10-7 | WT | WT | WT | WT | WT | WT |
| 8 | VICC-1 | p.V600K; c.1798_1799GT>AA | WT | WT | WT | WT | WT |
| 9 | VICC-2 | p.V600K; c.1798_1799GT>AA | WT | WT | WT | WT | WT |
| 10 | VICC-3 | p.V600E; c.1799T>A | WT | WT | WT | WT | WT |
| 11 | VICC-4 | p.V600E; c.1799T>A | WT | WT | WT | WT | WT |
| 12 | VICC-5 | p.V600R; c.1798_1799GT>AG | WT | WT | WT | WT | WT |
| 13 | VICC-6 | p.V600E; c.1799T>A | WT | WT | WT | WT | WT |
| 14 | VICC-7 | WT | WT | WT | WT | WT | WT |
| 15 | VICC-8 | WT | p.G13D; c.38G>A | WT | WT | WT | WT |
| 16 | VIC -9 | p.V600E; c.1799T>A | WT | WT | WT | WT | WT |
| 17 | VICC-10 | WT | WT | WT | WT | WT | WT |
| 18 | VICC-11 | WT | WT | WT | WT | WT | WT |

^a^WT; wild type
