# Supplementary material for: Routine Multiplex Mutational Profiling of Melanomas Enables Enrollment in Genotype-Driven Therapeutic Trials
Source: PLoS One. 2012 Apr 20;7(4):e35309. doi: 10.1371/journal.pone.0035309 (PMC3335021; doi:10.1371/journal.pone.0035309)
Supplement: Table S10 — SNaPshot assay results for the first 150 clinically screened melanomas. (DOCX) [file pone.0035309.s014.docx]

**Table S10**. SNaPshot assay results for the first 150 clinically screened melanomas.

| **Pt #^a^** | **Gender** | **Stage** | **Location of Primary Tumor** | **BRAF** | **CTNNB1** | **GNA11** | **GNAq** | **KIT** | **NRAS** | **Enrolled on clinical trial?** | **Genotype driven trial?** |
| --- | --- | --- | --- | --- | --- | --- | --- | --- | --- | --- | --- |
| 1 | Male | T2aN0M0 | Skin, arm | No mutation | No mutation | No mutation | No mutation | No mutation | Q61L |  |  |
| 2 | Male | TxNxM1c | Skin, back | No mutation | No mutation | No mutation | No mutation | No mutation | Q61R |  |  |
| 3 | Female | TxNxM1c | Skin, back | V600E | No mutation | No mutation | No mutation | No mutation | No mutation | Yes |  |
| 4 | Male | T4NxM0 | Oral mucosa | No mutation | No mutation | No mutation | No mutation | No mutation | No mutation |  |  |
| 5 | Male | TxNxM1c | Skin, back | No mutation | No mutation | No mutation | No mutation | No mutation | No mutation | Yes |  |
| 6 | Male | T1aN1bM0 | Skin, leg | No mutation | No mutation | No mutation | No mutation | No mutation | Q61R |  |  |
| 7 | Male | TxNxM1c | Skin, back | V600E | No mutation | No mutation | No mutation | No mutation | No mutation |  |  |
| 8 | Male | T1aN0M0 | Skin, leg | V600E | No mutation | No mutation | No mutation | No mutation | No mutation |  |  |
| 9 | Male | TxNxM1c | Skin, shoulder | V600E | No mutation | No mutation | No mutation | No mutation | No mutation | Yes | Yes |
| 10 | Male | TxNxM1c | Skin, neck | No mutation | No mutation | No mutation | No mutation | No mutation | No mutation |  |  |
| 11 | Male | TxNxM1c | Scalp | No mutation | No mutation | No mutation | No mutation | No mutation | No mutation |  |  |
| 12 | Male | T4aN3M1b | Skin, shoulder | No mutation | No mutation | No mutation | No mutation | No mutation | Q61K |  |  |
| 13 | Male | TxNxM1c | Skin, back | V600E | No mutation | No mutation | No mutation | No mutation | No mutation | Yes | Yes |
| 14 | Male | T4xN3M0 | Conjunctiva | V600E | No mutation | No mutation | No mutation | No mutation | No mutation |  |  |
| 15 | Female | TxNxM1c | Skin, leg | V600E | No mutation | No mutation | No mutation | No mutation | No mutation | Yes | Yes |
| 16 | Male | T2bN2cM0 | Skin, foot | No mutation | No mutation | No mutation | No mutation | No mutation | No mutation |  |  |
| 17 | Male | TxNxM1c | Skin, chest | No mutation | No mutation | No mutation | No mutation | No mutation | No mutation |  |  |
| 18 | Male | T4bN1M0 | Skin, shoulder | No mutation | No mutation | No mutation | No mutation | No mutation | Q61L |  |  |
| 19 | Male | TxNxM1c | Skin, arm | No mutation | No mutation | No mutation | No mutation | No mutation | G13R | Yes | Yes |
| 20 | Male | TxNxM1b | Skin, face | V600K | No mutation | No mutation | No mutation | No mutation | No mutation | Yes | Yes |
| 21 | Female | TxNxM1c | Skin, leg | V600E | No mutation | No mutation | No mutation | No mutation | No mutation |  |  |
| 22 | Male | TxNxM1a | Unknown | No mutation | No mutation | No mutation | No mutation | No mutation | Q61R |  |  |
| 23 | Female | TxNxM1b | Vulvar | No mutation | No mutation | No mutation | No mutation | L576P | No mutation | Yes | Yes |
| 24 | Male | TxNxM1b | Scalp | No mutation | No mutation | No mutation | No mutation | No mutation | No mutation | Yes |  |
| 25 | Male | T3aNxM0 | Skin, back | No mutation | No mutation | No mutation | No mutation | No mutation | No mutation |  |  |
| 26 | Female | T1aNxM0 | Skin, leg | No mutation | No mutation | No mutation | No mutation | No mutation | No mutation |  |  |
| 27 | Female | TxNxM1c | Unknown | No mutation | No mutation | No mutation | No mutation | No mutation | No mutation |  |  |
| 28 | Male | TxNxM1b | Scalp | No mutation | No mutation | No mutation | No mutation | No mutation | No mutation |  |  |
| 29 | Male | pT3bN0M0 | Skin, shoulder | No mutation | No mutation | No mutation | No mutation | No mutation | No mutation |  |  |
| 30 | Female | TxNxM1a | Skin, leg | No mutation | No mutation | No mutation | No mutation | No mutation | Q61R |  |  |
| 31 | Female | TxN3M0 | Unknown | V600E | No mutation | No mutation | No mutation | No mutation | No mutation |  |  |
| 32 | Male | T1aN3M0 | Skin, back | V600R | No mutation | No mutation | No mutation | No mutation | No mutation |  |  |
| 33 | Male | T1bN2cM0 | Scalp | V600M | No mutation | No mutation | No mutation | No mutation | No mutation |  |  |
| 34 | Male | T1bNxM0 | Skin, face | No mutation | No mutation | No mutation | No mutation | No mutation | No mutation |  |  |
| 35 | Male | T3aN0M0 | Skin, neck | No mutation | No mutation | No mutation | No mutation | No mutation | No mutation |  |  |
| 36 | Male | pT1aNxM0 | Skin, back | No mutation | No mutation | No mutation | No mutation | No mutation | Q61R |  |  |
| 37 | Male | TxNxM1c | Skin, foot | No mutation | No mutation | No mutation | No mutation | No mutation | Q61R | Yes | Yes |
| 38 | Female | T2aN0M0 | Skin, arm | No mutation | No mutation | No mutation | No mutation | No mutation | No mutation |  |  |
| 39 | Male | TxNxM1c | Skin, back | No mutation | No mutation | No mutation | No mutation | No mutation | No mutation | Yes |  |
| 40 | Male | T3aN0M0 | Skin, back | V600E | No mutation | No mutation | No mutation | No mutation | No mutation |  |  |
| 41 | Female | T2aN0M0 | Skin, leg | V600E | No mutation | No mutation | No mutation | No mutation | No mutation |  |  |
| 42 | Female | T3aN1M0 | Skin, back | V600E | No mutation | No mutation | No mutation | No mutation | No mutation |  |  |
| 43 | Male | T1bN0M0 | Skin, back | V600M | No mutation | No mutation | No mutation | No mutation | No mutation |  |  |
| 44 | Male | T4aN3M0 | Skin, shoulder | V600E | No mutation | No mutation | No mutation | No mutation | No mutation |  |  |
| 45 | Female | T1aN0M0 | Skin, back | No mutation | No mutation | No mutation | No mutation | L576P | No mutation |  |  |
| 46 | Male | T1bN0M0 | Subungual | No mutation | No mutation | No mutation | No mutation | No mutation | No mutation |  |  |
| 47 | Male | T4aN2cM1c | Skin, neck | No mutation | No mutation | No mutation | No mutation | No mutation | No mutation |  |  |
| 48 | Male | T1bN0M0 | Nose (nasoalar groove) | No mutation | No mutation | No mutation | No mutation | No mutation | No mutation |  |  |
| 49 | Female | TxNxM1c | Skin, chest | V600E | No mutation | No mutation | No mutation | No mutation | No mutation |  |  |
| 50 | Male | T4bN1bM0 | Skin, hand | No mutation | No mutation | No mutation | No mutation | L576P | No mutation |  |  |
| 51 | Male | T2aN0M0 | Skin, arm | No mutation | No mutation | No mutation | No mutation | No mutation | G12D |  |  |
| 52 | Male | TxNxM1c | Skin, back | No mutation | No mutation | No mutation | No mutation | No mutation | No mutation |  |  |
| 53 | Female | T1bN0M0 | Skin, arm | V600E | No mutation | No mutation | No mutation | No mutation | No mutation |  |  |
| 54 | Male | TxNxM1c | Skin, foot | No mutation | No mutation | No mutation | No mutation | No mutation | No mutation |  |  |
| 55 | Female | TxNxM1c | Unknown | V600E | No mutation | No mutation | No mutation | No mutation | No mutation |  |  |
| 56 | Male | TxNxM1b | Skin, back | V600E | No mutation | No mutation | No mutation | No mutation | No mutation |  |  |
| 57 | Female | T4bN1bM0 | Skin, hand | No mutation | No mutation | No mutation | No mutation | No mutation | No mutation |  |  |
| 58 | Male | T4nN3M0 | Skin, face | V600K | No mutation | No mutation | No mutation | No mutation | No mutation |  |  |
| 59 | Female | T3aN0M0 | Skin, hand | No mutation | S37F | No mutation | No mutation | No mutation | No mutation |  |  |
| 60 | Female | T1aN0M0 | Skin, foot | No mutation | No mutation | No mutation | No mutation | No mutation | No mutation |  |  |
| 61 | Male | T2bN0M0 | Skin, arm | No mutation | No mutation | No mutation | No mutation | No mutation | No mutation |  |  |
| 62 | Female | T1aN1bM0 | Skin, arm | No mutation | No mutation | No mutation | No mutation | No mutation | No mutation |  |  |
| 63 | Male | T4bN1bM0 | Scalp | No mutation | No mutation | No mutation | No mutation | No mutation | No mutation |  |  |
| 64 | Female | TxNxM1c | Unknown | No mutation | No mutation | No mutation | No mutation | No mutation | Q61R |  |  |
| 65 | Male | T1bN0M0 | Skin, back | No mutation | No mutation | No mutation | No mutation | No mutation | No mutation |  |  |
| 66 | Male | TxNxM1c | Skin, abdomen | No mutation | No mutation | No mutation | No mutation | No mutation | No mutation |  |  |
| 67 | Female | T4bN1bM1b | Rectum | No mutation | No mutation | No mutation | No mutation | No mutation | No mutation |  |  |
| 68 | Male | TxNxM1c | Skin, face | V600E | No mutation | No mutation | No mutation | No mutation | No mutation | Yes |  |
| 69 | Male | TxNxM1c | Ocular | No mutation | No mutation | No mutation | No mutation | No mutation | No mutation |  |  |
| 70 | Male | TxNxM1c | Skin, foot | V600E | No mutation | No mutation | No mutation | No mutation | No mutation |  |  |
| 71 | Male | T3N1bM0 | Skin, foot | V600E | No mutation | No mutation | No mutation | No mutation | No mutation |  |  |
| 72 | Female | TxNxM1c | Skin, arm | V600E | No mutation | No mutation | No mutation | No mutation | No mutation |  |  |
| 73 | Female | TisN3M0 | Skin, arm | No mutation | No mutation | No mutation | No mutation | No mutation | No mutation |  |  |
| 74 | Female | T2aN0M0 | Skin, back | V600E | No mutation | No mutation | No mutation | No mutation | No mutation |  |  |
| 75 | Male | TxNxM1c | Skin, shoulder | No mutation | No mutation | No mutation | No mutation | No mutation | No mutation | Yes |  |
| 76 | Female | T2bN3M0 | Scalp | V600R | No mutation | No mutation | No mutation | No mutation | No mutation |  |  |
| 77 | Female | T4bN0M0 | Anus | No mutation | No mutation | No mutation | No mutation | No mutation | No mutation |  |  |
| 78 | Male | TxNxM1a | Skin, arm | V600E | No mutation | No mutation | No mutation | No mutation | No mutation |  |  |
| 79 | Female | T2aN0M0 | Skin, arm | V600E | No mutation | No mutation | No mutation | No mutation | No mutation |  |  |
| 80 | Male | TxNxM1c | Skin, back | No mutation | No mutation | No mutation | No mutation | No mutation | No mutation |  |  |
| 81 | Male | TxNxM1c | Unknown | No mutation | No mutation | No mutation | No mutation | No mutation | No mutation |  |  |
| 82 | Male | TxNxM1c | Skin, back | No mutation | No mutation | No mutation | No mutation | No mutation | Q61R |  |  |
| 83 | Female | TxNxM1b | Skin, back | No mutation | No mutation | No mutation | No mutation | No mutation | No mutation | Yes |  |
| 84 | Male | TxNxM1c | Ocular | No mutation | No mutation | No mutation | Q209L | No mutation | No mutation | Yes | Yes |
| 85 | Female | TxNxM1c | Unknown | V600K | No mutation | No mutation | No mutation | No mutation | No mutation | Yes | Yes |
| 86 | Male | T4N0M1b | Sinus | No mutation | No mutation | No mutation | No mutation | No mutation | Q61H | Yes | Yes |
| 87 | Female | TxNxM1c | Skin, leg | V600E | No mutation | No mutation | No mutation | No mutation | No mutation | Yes | Yes |
| 88 | Female | TxNxM1c | Skin, arm | V600E | No mutation | No mutation | No mutation | No mutation | No mutation | Yes | Yes |
| 89 | Female | T1bN0M0 | Skin, face | V600E | No mutation | No mutation | No mutation | No mutation | No mutation |  |  |
| 90 | Female | T4bN3M0 | Skin, shoulder | V600K | No mutation | No mutation | No mutation | No mutation | No mutation |  |  |
| 91 | Female | T2xN0M0 | Skin, arm | No mutation | No mutation | No mutation | No mutation | No mutation | No mutation |  |  |
| 92 | Male | TxNxM1c | Skin, back | No mutation | No mutation | No mutation | No mutation | No mutation | No mutation |  |  |
| 93 | Female | TxNxM1b | Skin, face | No mutation | No mutation | No mutation | No mutation | No mutation | No mutation |  |  |
| 94 | Female | TxNxM1c | Sinus | No mutation | No mutation | No mutation | No mutation | No mutation | No mutation | Yes |  |
| 95 | Female | TxNxM1c | Ocular | No mutation | No mutation | No mutation | Q209L | No mutation | No mutation | Yes | Yes |
| 96 | Male | T1aN0M0 | Scalp | V600K | No mutation | No mutation | No mutation | No mutation | No mutation |  |  |
| 97 | Male | TxNxM1c | Unknown | V600E | No mutation | No mutation | No mutation | No mutation | No mutation | Yes | Yes |
| 98 | Male | T4aN3M0 | Skin, face | V600E | No mutation | No mutation | No mutation | No mutation | No mutation |  |  |
| 99 | Female | T2aN0M0 | Skin, abdomen | V600E | No mutation | No mutation | No mutation | No mutation | No mutation |  |  |
| 100 | Male | TxNxM1c | Skin, shoulder | V600K | No mutation | No mutation | No mutation | No mutation | No mutation |  |  |
| 101 | Male | T2aN0M0 | Skin, face | No mutation | No mutation | No mutation | No mutation | No mutation | No mutation |  |  |
| 102 | Male | T4bN3M0 | Skin, foot | No mutation | No mutation | No mutation | No mutation | No mutation | No mutation |  |  |
| 103 | Female | T4sinusN0M0 | Sinus | No mutation | No mutation | No mutation | No mutation | No mutation | No mutation |  |  |
| 104 | Female | T2aN0M0 | Skin, face | No mutation | No mutation | No mutation | No mutation | No mutation | No mutation |  |  |
| 105 | Male | TxN2bM0 | Unknown | V600E | No mutation | No mutation | No mutation | No mutation | No mutation |  |  |
| 106 | Female | T3bN1aM0 | Skin, neck | No mutation | No mutation | No mutation | No mutation | No mutation | No mutation |  |  |
| 107 | Male | TxNxM1b | Skin, shoulder | No mutation | No mutation | No mutation | No mutation | No mutation | G12D |  |  |
| 108 | Male | TxNxM1c | Skin, neck | V600E | No mutation | No mutation | No mutation | No mutation | No mutation | Yes | Yes |
| 109 | Female | pT4bN1aM0 | Skin, back | V600E | No mutation | No mutation | No mutation | No mutation | No mutation |  |  |
| 110 | Female | TxNxM1b | Skin, arm | No mutation | No mutation | No mutation | No mutation | No mutation | No mutation |  |  |
| 111 | Female | pT2aN1aM0 | Skin, back | V600E | No mutation | No mutation | No mutation | No mutation | No mutation |  |  |
| 112 | Male | TxNxM1c | unknown | No mutation | No mutation | No mutation | No mutation | No mutation | G12C |  |  |
| 113 | Male | TxNxM1c | Skin, neck | V600R | No mutation | No mutation | No mutation | No mutation | No mutation |  |  |
| 114 | Female | TxNxM1c | Skin, back | V600E | No mutation | No mutation | No mutation | No mutation | No mutation | Yes | Yes |
| 115 | Female | TxNxM1c | Skin, arm | No mutation | No mutation | No mutation | No mutation | No mutation | Q61L | Yes |  |
| 116 | Female | TxN3M0 | Skin, foot | No mutation | No mutation | No mutation | No mutation | No mutation | G12A |  |  |
| 117 | Male | T2aNxM0 | Scalp | No mutation | No mutation | No mutation | No mutation | No mutation | No mutation |  |  |
| 118 | Male | TxNxM1c | Skin, back | V600E | No mutation | No mutation | No mutation | No mutation | No mutation | Yes | Yes |
| 119 | Male | TxNxM1c | Unknown | V600E | No mutation | No mutation | No mutation | No mutation | No mutation |  |  |
| 120 | Male | T4bN2cM0 | Skin, foot | No mutation | No mutation | No mutation | No mutation | No mutation | No mutation |  |  |
| 121 | Male | T4bN0M0 | Skin, face | No mutation | No mutation | No mutation | No mutation | No mutation | No mutation |  |  |
| 122 | Male | TxNxM1a | Skin, shoulder | V600E | No mutation | No mutation | No mutation | No mutation | No mutation |  |  |
| 123 | Male | TxN2M0 | Unknown | V600E | No mutation | No mutation | No mutation | No mutation | No mutation |  |  |
| 124 | Male | T3bN2aM0 | Skin, leg | No mutation | No mutation | No mutation | Not reported | No mutation | No mutation |  |  |
| 125 | Male | TxNxM1b | Skin, face | No mutation | No mutation | No mutation | No mutation | No mutation | No mutation |  |  |
| 126 | Male | T4aN0M0 | Skin, face | No mutation | No mutation | No mutation | No mutation | No mutation | No mutation |  |  |
| 127 | Male | T2bN2cM0 | Skin, arm | No mutation | No mutation | No mutation | No mutation | No mutation | No mutation |  |  |
| 128 | Female | TxNxM1b | Skin, back | No mutation | S45P | No mutation | No mutation | No mutation | Q61L | Yes | Yes |
| 129 | Male | TxN3M0 | Skin, neck | No mutation | No mutation | No mutation | No mutation | No mutation | G13R |  |  |
| 130 | Female | TxNxM1c | Skin, back | V600K | No mutation | No mutation | No mutation | No mutation | No mutation | Yes | Yes |
| 131 | Male | TxNxM1c | Unknown | No mutation | No mutation | No mutation | Q209P | No mutation | No mutation |  |  |
| 132 | Male | T4bNxM0 | Nasal cavity | No mutation | No mutation | No mutation | No mutation | No mutation | Q61R |  |  |
| 133 | Male | TxNxM1c | Skin, shoulder | V600E | No mutation | No mutation | No mutation | No mutation | No mutation | Yes | Yes |
| 134 | Female | TxNxM1c | Skin, leg | No mutation | No mutation | No mutation | No mutation | No mutation | No mutation | Yes |  |
| 135 | Male | TxNxM1c | Ocular | No mutation | No mutation | No mutation | No mutation | No mutation | No mutation | Yes |  |
| 136 | Female | TxNxM1c | Ocular | No mutation | No mutation | No mutation | Q209L | No mutation | No mutation | Yes | Yes |
| 137 | Male | TxNxM1c | Skin, shoulder | V600E | No mutation | No mutation | No mutation | No mutation | No mutation |  |  |
| 138 | Male | T3aN3M0 | Skin, leg | No mutation | No mutation | No mutation | No mutation | No mutation | No mutation |  |  |
| 139 | Male | TxNxM1c | Skin, arm | V600E | No mutation | No mutation | No mutation | No mutation | No mutation |  |  |
| 140 | Female | T4uvealNxM1b | Ocular | No mutation | No mutation | No mutation | Q209P | No mutation | No mutation |  |  |
| 141 | Male | TxNxM1c | Skin, shoulder | No mutation | No mutation | No mutation | No mutation | No mutation | No mutation |  |  |
| 142 | Male | TxNxM1a | Skin, back | V600E | No mutation | No mutation | No mutation | No mutation | No mutation |  |  |
| 143 | Female | TxNxM1c | Unknown | V600E | No mutation | No mutation | No mutation | No mutation | No mutation |  |  |
| 144 | Female | TxNxM1c | Skin, back | No mutation | No mutation | No mutation | No mutation | No mutation | No mutation |  |  |
| 145 | Male | TxNxM1c | Unknown | No mutation | No mutation | No mutation | No mutation | No mutation | No mutation |  |  |
| 146 | Female | TxNxM1b | Vulvar | V600E | No mutation | No mutation | No mutation | No mutation | No mutation | Yes | Yes |
| 147 | Female | T4aN0M0 | Skin, arm | No mutation | No mutation | No mutation | No mutation | No mutation | No mutation |  |  |
| 148 | Male | TxNxM1c | Skin, leg | No mutation | No mutation | No mutation | No mutation | No mutation | Q61R | Yes | Yes |
| 149 | Female | TxNxM1c | Skin, leg | No mutation | No mutation | No mutation | No mutation | No mutation | G13R |  |  |
| 150 | Female | TxNxM1c | Ocular | No mutation | No mutation | No mutation | Q209P | No mutation | No mutation |  |  |

^a^pt; patient
