# Supplementary material for: Routine Multiplex Mutational Profiling of Melanomas Enables Enrollment in Genotype-Driven Therapeutic Trials
Source: PLoS One. 2012 Apr 20;7(4):e35309. doi: 10.1371/journal.pone.0035309 (PMC3335021; doi:10.1371/journal.pone.0035309)
Supplement: Table S11 — Open genotype-driven clinical trials at Vanderbilt University. (DOC) [file pone.0035309.s015.doc]

**Table S11**. Open genotype-driven clinical trials at Vanderbilt University.

| **Trial ID** | **Agent** | **Target** |
| --- | --- | --- |
| NCT01266967 | GSK2118436 | BRAF V600E or K |
| NCT10116980 | Vemurafenib | BRAF V600E |
| NCT01072175 | GSK2118436 + GSK1120212 | BRAF V600E or K + MEK |
| NCT01153763 | GSK2118436 | BRAF V600E or K |
| NCT10116980 | GSK1120212 | BRAF V600E or K (MEK) |
| NCT01271803 | Vemurafenib + GDC-0973 | BRAF V600E + MEK |
| NCT00827177 | ARQ197 + Sorafenib | NRAS |
| NCT0102822 | Nilotinib | KIT |
| NCT00577382 | Sunitinib | KIT |
| NCT00866177 | AZD6244 | GNAQ + GNA11 |
| NCT00948467 | TAK-733 | GNAQ + GNA11 |
